# Closed-loop electrical stimulation prevents focal epilepsy progression and long-term memory impairment

**DOI:** 10.1038/s41593-025-01988-1

**Published:** 2025-06-23

**Authors:** Jose J. Ferrero, Ahnaf R. Hassan, Zelin Yu, Zifang Zhao, Liang Ma, Cynthia Wu, Shan Shao, Takeshi Kawano, Judah Engel, Werner Doyle, Orrin Devinsky, Dion Khodagholy, Jennifer N. Gelinas

**Affiliations:** 1 Department of Neurology, Columbia University Irving Medical Center, New York City, NY USA; 2 Department of Pediatrics, University of California, Irvine, Irvine, CA USA; 3 Department of Electrical Engineering, Columbia University, New York City, NY USA; 4 Department of Electrical and Electronic Information Engineering, Toyohashi University of Technology, Toyohashi, Japan; 5 Institute for Research on Next-generation Semiconductor and Sensing Science (IRES2), Toyohashi University of Technology, Toyohashi, Japan; 6 Comprehensive Epilepsy Center, New York University, New York City, NY USA; 7 Department of Electrical Engineering and Computer Science, University of California, Irvine, Irvine, CA USA; 8 Department of Anatomy and Neurobiology, University of California, Irvine, Irvine, CA USA; 9 Children’s Hospital of Orange County, Orange, CA USA

**Keywords:** Epilepsy, Consolidation

## Abstract

Interictal epileptiform discharges (IEDs) are expressed in epileptic networks and disrupt cognitive functions. It is unclear whether addressing IED-induced dysfunction could improve epilepsy outcomes, as most therapeutic approaches target seizures. We show, in a kindling model of progressive focal epilepsy, that IEDs produce pathological oscillatory coupling associated with prolonged, hypersynchronous neural spiking in synaptically connected cortex and expand the brain territory capable of generating IEDs. A similar relationship between IED-mediated oscillatory coupling and temporal organization of IEDs across brain regions was identified in human participants with refractory focal epilepsy. Spatiotemporally targeted closed-loop electrical stimulation triggered on hippocampal IED occurrence eliminated the abnormal cortical activity patterns, preventing the spread of the epileptic network and ameliorating long-term spatial memory deficits in rodents. These findings suggest that stimulation-based network interventions that normalize interictal dynamics may be an effective treatment of epilepsy and its comorbidities, with a low barrier to clinical translation.

## Abstract (web summary)

Pathological neural communication drives the spread of the epileptic network and contributes to memory impairment in focal epilepsy. The authors show that closed-loop electrical stimulation in rodents can prevent this interaction and preserve long-term memory.

## Main

Focal epilepsies are associated with large-scale structural and functional neural network abnormalities that can extend beyond the brain regions responsible for seizure generation^1^. These alterations are associated with neuropsychiatric comorbidities that can worsen over time^2^. Epilepsy therapeutics focused on eliminating seizures have had limited efficacy in modifying disease course and addressing these comorbidities, which can profoundly impair quality of life^3–5^. Epileptic networks predominantly exist in the interictal state, which contains aberrant dynamics and epileptiform patterns that interfere with physiological processes^6,7^. Although certain antiseizure medications can decrease the occurrence of interictal epileptiform discharges (IEDs), their nonspecific inhibition of neuronal excitability is linked to adverse effects on information processing, impairing evaluation of IED burden on cognition^8^. IEDs have been associated with both epileptogenic and anti-ictogenic processes, suggesting variable effects depending on the network state^9^. Thus, it remains unclear whether and how altering interictal epileptiform activity could treat epilepsy and its comorbidities.

Continuous, intermittent deep brain stimulation and vagus nerve stimulation are aimed at impacting hubs of highly interconnected, complex networks in an empirical attempt to induce desynchronization and prevent seizure initiation^10,11^. Closed-loop electrical stimulation (such as we have used here, or responsive neurostimulation systems; NeuroPace RNS System) can be configured to deliver abortive stimulation to the seizure onset zone in response to detected ictal patterns^12–15^. In each case, seizure reductions emerge well after therapy onset, implicating chronic, plasticity-related mechanisms^16–18^. Furthermore, in animal epilepsy models, interventions that normalize interictal state dynamics can improve memory^19^. These results support that mechanistically driven manipulation of interictal patterns could gradually reshape neural networks to support physiological brain functions and suppress epileptic activity.

The potential target mechanisms to achieve these goals in epileptic networks are mostly unknown but could include amelioration of altered physiological interactions. During nonrapid eye movement (NREM) sleep, consolidation of episodic memory requires precise correlation of hippocampal (HC) and cortical oscillations, including hippocampal sharp wave-ripples, the cortical slow oscillation, cortical spindles and cortical ripples^20–23^. IEDs, a key pathological output of the interictal state, disrupt these critical interactions by initiating strong, precise temporal coupling with spindles, which surpasses physiological ripple–spindle correlation^24^. IED–spindle coupling occurs in rodent models and human patients with focal epilepsy, establishing this phenomenon as a potential interictal therapeutic target^25–27^.

Here we show that this IED–spindle coupling drives prolonged, hypersynchronous cortical spiking and predisposes to the generation of local cortical IEDs, effectively spreading the epileptic network. Our data support that a similar process may establish independent foci of interictal epileptiform activity in patients with focal epilepsy. Cortical closed-loop electrical stimulation that inhibits IED–spindle coupling can prevent expression of cortical IEDs, mitigating enlargement of the epileptic network and preserving long-term memory in a rodent model of focal epilepsy. These results support the use of spatiotemporally focused, interictal-based approaches to normalize epileptic activity patterns and address cognitive comorbidities.

## Results

### Hippocampal IEDs create an independent cortical IED focus

**Fig. 1:**
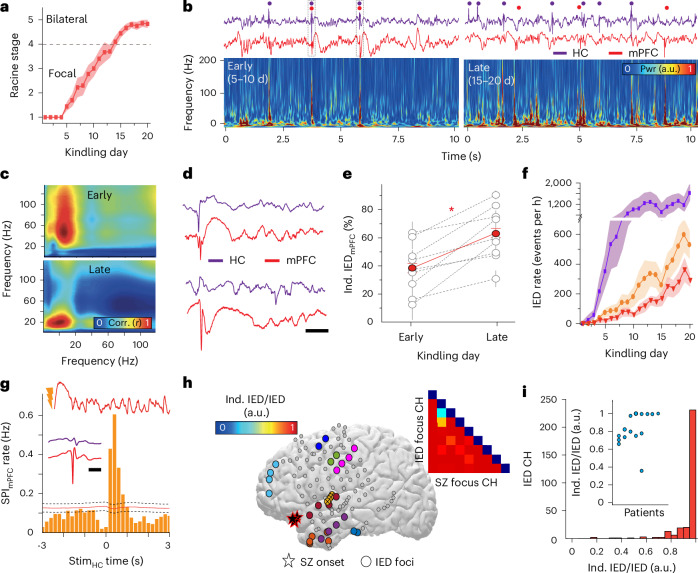
Progression of focal epilepsy is associated with the creation of an independent focus of interictal epileptic activity. **a**, Average Racine stage progression across kindling (*n* = 10 rats). **b**, Sample LFP traces acquired simultaneously from HC and mPFC. Dots indicate time of IED (top), with corresponding mPFC spectrogram (bottom) for early and late stages of kindling. **c**, Sample comodulograms demonstrating decreased cross-frequency coupling between hippocampus and mPFC during progression from early (top) to late stage (bottom) of kindling. **d**, Sample LFP traces from HC and mPFC, showing IED_HC_ coupled (top) and independent IED_mPFC_ (bottom). Scale bar, 100 ms. **e**, Increase in the percentage of independent mPFC IEDs from early to late stage of kindling (*n* = 10 rats, 37 early- to 37 late-stage sessions; unpaired two-tailed *t*-test = −5.34, *P* = 1.03 × 10^−9^). *P < 0.05. **f**, Occurrence of IEDs in hippocampus (purple) and mPFC (total, orange; independent, red) in NREM sleep over kindling (*n* = 10 rats). **g**, CCG of a-IED_HC_ with detected mPFC spindles (bottom) during NREM sleep (95% confidence intervals with midpoints represented as black dashed and red lines, respectively; *n* = 2,429 spindles, 3,658 stimulations, one sample unkindled rat). Inset, sample mPFC trace after pulse stimulation showing evoked spindle oscillation (top). Independent mPFC (red) IED and HC traces (purple; bottom, scale bar—200 ms). **h**, Spatial representation of clinically identified IED foci (one color per focus) and seizure onset zone (stars; *n* = 1 sample human participant). Inset demonstrates the ratio of IEDs in each focus that are independent (>100 ms apart) from seizure onset zone IEDs to total IEDs in the focus (independent IED ratio). Columns represent seizure onset zone channels, and rows represent IED focus channels. **i**, Histogram of the ratio of independent IEDs to all IEDs across all channels (*n* = 9 human participants). Inset demonstrates the independent IED ratio across all clinically identified IED foci (*n* = 9 human participants). **a**–**i**, Data are presented as mean ± s.e.m. Source data

To examine how hippocampal–cortical network interactions were altered in the presence of ongoing, progressive epileptic activity, we used hippocampal kindling in freely moving rats while acquiring in vivo electrophysiology data from hippocampus and a key synaptically connected cortical area, medial prefrontal cortex (mPFC). After baseline recording sessions in each animal, we extended an established protocol^24^ to permit more rapid transition from focal hippocampal to bilateral convulsive seizures, with rats consistently advancing to Racine stage 4 after 10–15 days of kindling (Fig. 1a and Supplementary Fig. 1a). During NREM sleep, but not across other behavioral states (rapid eye movement (REM) and wakefulness), we observed a shift in the coherence of hippocampal–mPFC activity across the kindling protocol (Supplementary Fig. 1b,c). Early kindling (days 5–10) was associated with increased coherence relative to baseline, but with further progression of kindling (late kindling, days 15–20), coherence decreased (Fig. 1b,c and Supplementary Fig. 1b). Close examination of mPFC waveform and spectral properties suggested that spontaneous, hippocampal-independent waveforms meeting criteria for IEDs began to occur in the mPFC during late kindling (Fig. 1d). To investigate this possibility, we detected IEDs separately in hippocampus and mPFC and determined their temporal relationship. mPFC IEDs occurring >100 ms from a hippocampal IED were designated as independent from the hippocampus, as IED waveforms were fully nonoverlapping at this interval. Such independent mPFC IEDs were detected in all rats, with incidence higher at the later compared to earlier kindling stages (Fig. 1e). Hippocampal IED occurrence rate demonstrated an early, rapid increase, whereas total and independent mPFC IEDs emerged at a delay, with occurrence accelerating only late in kindling (Fig. 1f; polynomial fitting—HC, *y* = 7.67–15.52*x* + 16.87*x*^2^ + 0.15*x*^3^; mPFC_all_, *y* = 8.18–11.70*x* + 4.23*x*^2^ + 0.026*x*^3^; mPFC_ind_, *y* = 2.91–3.59*x* + 1.29*x*^2^ + 0.011*x*^3^. HC versus mPFC_all_, *F* = 61.34, *P* = 1.49 × 10^−14^; HC versus mPFC_ind_, *F* = 91.59, *P* = 0; mPFC_all_ versus mPFC_ind_, *F* = 27.20, *P* = 6.79 × 10^−10^; Supplementary Fig. 2a). Occurrence of independent mPFC IEDs was further supported by lack of evidence for any longer latency interactions between hippocampal and mPFC IEDs. Hippocampal spectrograms trigger-averaged on the occurrence time of mPFC IEDs demonstrated a broadband, high-power transient in the hippocampal local field potential (LFP), consistent with a temporally precise co-occurring hippocampal IED in early kindling, with decreased power in late kindling (Supplementary Fig. 2b). Similarly, cross-correlation of the separately detected hippocampus and mPFC IEDs revealed a significant peak of co-occurrence only within 100 ms. In late kindling, although the IED co-occurrence was decreased, no additional significant peaks of correlation were present (Supplementary Fig. 2c). Because the occurrence of hippocampal transients, including sharp wave-ripples and IEDs, is highly bilaterally synchronous in this model (Supplementary Fig. 2d,e; ref. ^28^), the contralateral hippocampus could not be the actual source of the putative independent mPFC IEDs either. Taken together, these results indicate that the progression of epileptic activity in the hippocampus reliably leads to the expression of IEDs in the mPFC. These mPFC IEDs eventually lose dependence on hippocampal IEDs, suggesting that the mPFC has become an independent focus of interictal epileptic activity.

We hypothesized that the emergence of these independent mPFC IEDs was enabled by the repetitive, pathological input received from the hippocampus in the form of hippocampal IEDs. We tested this hypothesis by using repeated (5 s intervals) single pulse stimulation to the hippocampal commissure, a protocol capable of inducing artificial hippocampal IEDs (a-IED_HC_) that resemble spontaneous hippocampal IEDs^29^ (Supplementary Fig. 3a). The interval between a-IED_HC_ stimulation was based on the mean inter-IED interval observed in kindled rats (Supplementary Fig. 3b). Application of this protocol to normal rats, in the absence of any kindling, initially resulted in a small, monosynaptic latency-evoked response in the mPFC as well as prominent generation of a temporally coupled spindle oscillation (Fig. 1g and Supplementary Fig. 3c). Prolonged daily administration of a-IED_HC_ resulted in detection of mPFC IEDs that resembled those identified in kindled rats (Supplementary Fig. 3d,e) and occurred in the absence of concurrent hippocampal IEDs (Supplementary Fig. 3f,g). Thus, the ongoing occurrence of hippocampal IEDs is sufficient to pathologically modulate the mPFC such that it becomes capable of generating independent IEDs.

These results suggested a link to interictal networks of patients with refractory focal epilepsy, who often display multiple interictal epileptiform foci and IED–spindle coupling^26,30^. We examined sleep intracranial electroencephalography (iEEG) from patients undergoing large-scale electrophysiological monitoring in their presurgical evaluation with multiple IED foci. These patients expressed two to nine IED foci, one of which overlapped with the clinically determined seizure onset zone in each participant (Fig. 1h). IEDs at foci outside of the seizure onset zone had a variable temporal relationship with IEDs in the seizure onset zone, with 0–64% co-occurrence (Fig. 1i). Thus, independent IED foci commonly develop in refractory focal human epilepsy.

### Hippocampal–cortical coupling links to independent mPFC IEDs

**Fig. 2:**
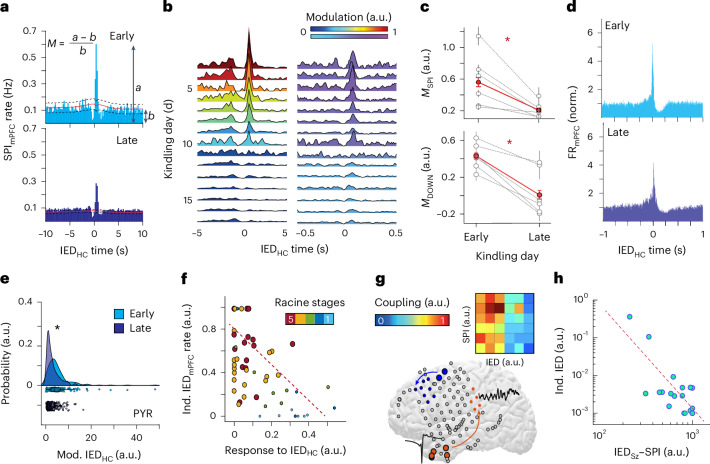
Pathological hippocampal–cortical dynamics link to independent IED foci. **a**, Sample CCGs of IED_HC_ and spindles at early (top) and late (bottom) kindling (early = 1,182/5,737; late = 10,875/4,881 IEDs/SPI). The 95% confidence intervals with midpoints represented as black dashed and red lines, respectively. **b**, Longitudinal modulation of IED_HC_–spindle (right) and hippocampal IED_HC_–SO (left) coupling across kindling from a representative rat. **c**, Decrease in coupling modulation (M) from early to late stage of kindling, for IED_HC_–spindle coupling (top; unpaired, two-tailed *t*-test, 33 early and 29 late sessions, *n* = 6 rats, *t* = 5.74, *P* = 9.3910^−7^) and IED_HC_–SO coupling (bottom; unpaired, two-tailed *t*-test, 33 early and 29 late sessions, *n* = 6 rats, *t* = 7.41, *P* = 1.31 × 10^−9^). Data are presented as mean ± s.e.m. *P < 0.05. **d**, Histogram of clustered mPFC neurons at the time of IED_HC_ for early (top, *n* = 69 neurons) and late kindling (*n* = 110 neurons from sample rat). **e**, IFR probability distribution of significantly modulated (16.63%) mPFC pyramidal neurons at the time of IED_HC_ during early and late kindling (unpaired, two-tailed *t*-test, *t*  = 5.28, *P* = 1.00 × 10^−7^, *n* = 359 neurons). *P < 0.05. **f**, Relationship between responsiveness to IED_HC_ (composite scored based on coupling of IED_HC_ with SPI_mPFC_, SO_mPFC_ and MUA_mPFC_) and rate of independent IED_mPFC_ (*n* = 57 sessions from six rats; color code indicates Racine stage with dashed line showing pairwise linear correlation; two-tailed Pearson *P* = 2.80 × 10^−5^). **g**, Spatial representation of IED–SPI coupling for two sample IED foci (orange and blue; large circles are IED focus and small circles are maximal region of SPI coupling) from one sample human participant. Inset demonstrates the amount of significant IED–SPI coupling occurring between each IED focus; each column represents an IED focus (*n* = 6 IED foci from one sample participant). **h**, Relationship between strength of SPI coupling modulation to seizure onset zone IEDs and rate of IEDs independent from those at seizure onset zone (*n* = 9 participants). Coupling strengths and IED rates are normalized within participants (two-tailed Spearman *r* = 0.78; *P* = 3.30 × 10^−7^). SO, slow oscillation. Source data

What mediates the emergence of this independent cortical interictal epileptic activity? We established that hippocampal IEDs can reset the phase of the mPFC slow oscillation and induce precisely timed spindles^12,24,26,27,31–33^ (Fig. 1h and Supplementary Fig. 4a). We hypothesized that this pathological oscillatory interaction has a critical role in the process. Therefore, we examined the relationship between hippocampal IEDs, mPFC slow oscillation and mPFC spindle oscillations at a timepoint characterized by sparse independent mPFC IEDs (kindling days 5–10) as compared to a timepoint with frequent, prominent mPFC IEDs (kindling days 15–20). Hippocampal IEDs displayed a strong, significant correlation with mPFC spindles (Fig. 2a, top), corroborated by prominent spindle power during this epoch (Supplementary Fig. 4b–d). Unexpectedly, this IED–spindle coordination, although still significant, decreased in magnitude during later kindling stages (Fig. 2a–c). Similarly, there was a progressive diminution of coupled slow oscillations in the mPFC to incoming hippocampal IEDs across kindling (Fig. 2b,c). We investigated whether this change could be related to the amplitude of hippocampal IEDs. Hippocampal IED amplitudes increased from early to late kindling (Supplementary Fig. 5a–c), indicating that hippocampal IED potency was not driving the observed change in hippocampal–cortical interactions. mPFC IED amplitudes also increased with kindling, and independent mPFC IEDs were similar in amplitude compared to hippocampal-dependent mPFC IEDs (Supplementary Fig. 5d–g). These results suggest the potential for an mPFC plasticity process (though presynaptic hippocampal plasticity remained a possibility).

To investigate this notion, we analyzed single neuron activity in the mPFC, classifying clustered units as putative pyramidal cells and interneurons based on established waveform and firing properties (*n* = 2,733 units from *n* = 6 rats)^34^. Hippocampal IEDs initially induced a strong increase in mPFC neural spiking at milliseconds latency, but this responsiveness significantly decreased at the later kindling timepoint (Fig. 2d,e and Supplementary Fig. 6). This decrease was predominantly mediated by diminished pyramidal cell spiking (Fig. 2e and Supplementary Fig. 7), although the temporal precision of both hippocampal IED-generated pyramidal cell and interneuron spiking was also reduced (Supplementary Fig. 8a,b). Local, independent mPFC IEDs could theoretically cause a brief (approximately 200 ms) decreased capacity of mPFC neurons to respond to incoming hippocampal IEDs due to post-IED hyperpolarization. To ensure that the decreased mPFC responsiveness to hippocampal IEDs was not due to such hyperpolarized epochs, we removed instances in which mPFC IEDs preceded hippocampal IEDs within 200 ms (<1% of hippocampal IEDs) and found that our results were unchanged. In parallel, mPFC pyramidal cells and interneurons increased their spiking during independent mPFC IEDs across kindling (Supplementary Fig. 8c–f). Furthermore, we found that there was a significant negative correlation between mPFC oscillatory and neural spiking responsiveness to hippocampal IEDs and expression of independent mPFC IEDs across rats. These features were additionally able to differentiate the capacity of the epileptic network to generate focal versus bilateral convulsive seizures (Fig. 2f and Supplementary Fig. 9). Together, these results suggest that mPFC adaptively modulates its activity patterns as hippocampal epilepsy progresses, downregulating output triggered by the epileptic focus but enhancing local interictal epileptic activity.

We next asked whether a similar phenomenon could occur in human participants with focal epilepsy. Because large-scale, long-duration iEEG monitoring is not typically available in human participants, we examined the relationship between IED–spindle coupling and expression of independent IEDs across the participants’ multiple clinical IED foci. Clinical IED foci were associated with distinct spatiotemporal patterns of IED–spindle coupling that variably involved other IED foci (Fig. 2g). Thus, for each clinical IED focus, we quantified the degree to which this brain region expressed spindles temporally coupled to IEDs in the seizure onset zone and the rate of IEDs occurring independently from those in the seizure onset zone. We found a strong negative correlation between these two measures (Fig. 2h). These results suggest that oscillatory coupling responsiveness decreases as IED foci become more independent from the seizure onset zone.

### Hippocampal IEDs induce hypersynchronous cortical activity

**Fig. 3:**
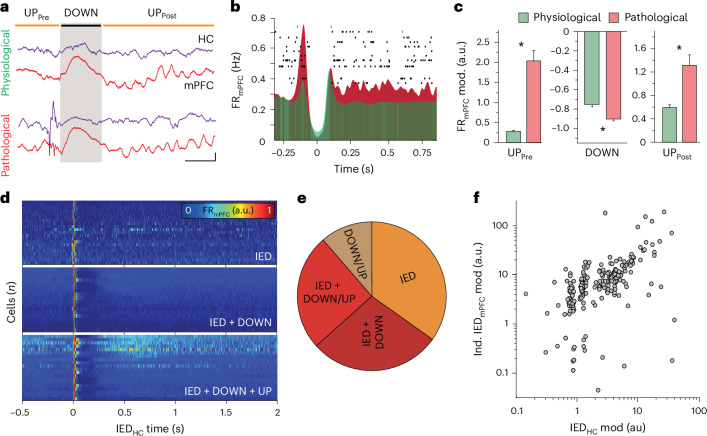
Hypersynchronous mPFC neuronal recruitment into pathological oscillatory sequences primes generation of independent mPFC IED-related neural spiking. **a**, Sample LFP traces from HC and mPFC demonstrating physiological (top) and pathological (bottom) transition through cortical ‘UP’ and ‘DOWN’ states (scale bar, 500 μV, 200 ms). **b**, Representative raster plot of mPFC neural spiking (*n* = 13 sample neurons) overlaid on averaged peri-event firing rate histogram of mPFC neurons during physiological and pathological transition through cortical ‘UP’ and ‘DOWN’ states (*n* = 351 neurons from one sample rat). **c**, Differences in mPFC neural firing rate modulation for physiological and pathological transition through cortical ‘UP’ and ‘DOWN’ states—UP_pre_ (left; averaged normalized values for 100 ms preceding cortical ‘DOWN’: Mann–Whitney test, *U* = 2,289, *P* = 5.10 × 10^−19^), DOWN (middle; minimum normalized values for 200 ms following onset of cortical ‘DOWN’ state, *U* = 11219, *P* = 4.44 × 10^−16^), UP_post_ (right; averaged normalized values for 500 ms after peak of cortical ‘DOWN’ state, *U* = 5,015, *P* = 1.85 × 10^−4^). Data are presented as mean ± s.e.m., and all tests are unpaired, two-tailed with *n* = 118 sessions. *P < 0.05. **d**, Representative normalized firing rate heatmap for mPFC neurons modulated by (1) hippocampal IEDs (top), (2) IEDs and pathological coupled cortical ‘DOWN’ state (middle) and (3) IEDs and pathological coupled cortical ‘DOWN’ and ‘UP’ states (bottom); *n* = 20 most highly modulated neurons per category, derived from five rats. **e**, Proportion of mPFC neurons modulated by categories of pathological events (*n* = 617 neurons modulated by pathological events (IED, or pathological IED-induced ‘DOWN/UP’ state) of total 2,733 clustered mPFC neurons from six rats). IED only—34.8%; IED + cortical ‘DOWN’ state—28.4%; IED + cortical ‘DOWN/UP’—25.3%; cortical ‘DOWN’ + cortical ‘UP’ only—11.5%). **f**, Relationship between mPFC neural firing rate modulation during hippocampal IEDs and independent mPFC IEDs (*n* = 323 neurons, six rats; two-tailed Spearman *ρ* = 0.52; *P* = 3 × 10^−24^). Source data

We sought to understand the mechanisms of IED-induced modulation at the neuronal level. In kindled rats, we studied how mPFC neurons responded to a hippocampal IED relative to a comparable physiological epoch. Because hippocampal IEDs reset the cortical slow oscillation phase before coupled spindle induction (Supplementary Fig. 4a), the resulting pattern resembles the transition from one cortical ‘UP’ state (UP_pre_), through a cortical ‘DOWN’ state, to a subsequent ‘UP’ state (UP_post_; Fig. 3a), which is often associated with physiological hippocampal–cortical communication. We identified cortical ‘DOWN’ to ‘UP’ state transitions in kindled rats and classified them as either (1) pathological (triggered by a hippocampal IED in UP_pre_ within 200 ms of the ‘DOWN’ state) or (2) physiological (absence of a hippocampal IED; Fig. 3a,b). We also identified physiological transitions in the baseline state before initiation of kindling. Pathological ‘UP’ states (UP_pre_ and UP_post_) were characterized by increased mPFC population spiking compared to physiological ‘UP’ states from both baseline and kindled sessions, and the pathological ‘DOWN’ state had a more profound decrease in spiking (Fig. 3c and Supplementary Fig. 10a). These changes were mediated by both pyramidal cells and interneurons (Supplementary Fig. 10b) and were emerging before the independent mPFC IEDs became frequent (Supplementary Fig. 10c), suggesting that hippocampal IEDs can induce hypersynchronous mPFC spiking that extends hundreds of milliseconds beyond the initial hippocampal–cortical synaptic interaction and potentially primes the mPFC for subsequent expression of local pathological activity patterns.

We next investigated the firing patterns of individual mPFC neurons during these epochs. In total, 22.5% of mPFC neurons exhibited significant firing rate modulation with incoming hippocampal IEDs and/or the evoked pathological DOWN and UP states (Fig. 3d,e). Only a small proportion of cells were modulated by the pathological cortical DOWN or UP state without any hippocampal IED-related modulation, indicating that a core group of hippocampal-responsive mPFC neurons was responsible for the prolonged hypersynchronous mPFC population patterns. Furthermore, this neuronal core was involved in generating independent mPFC IEDs. There was a significant correlation between the strength of hippocampal IED- and independent mPFC IED-driven firing rate modulation across individual neurons (Fig. 3f). The dual participation of these mPFC neurons suggests a relationship between hippocampal-responsive patterns and the origin of independent mPFC IEDs.

### Closed-loop mPFC stimulation prevents IED-related activity

**Fig. 4:**
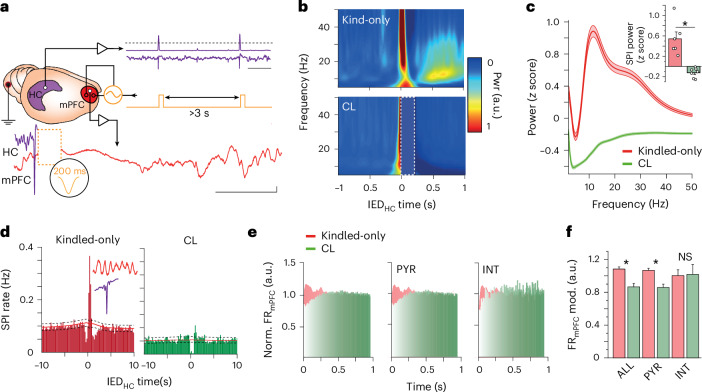
CL IED_HC_-triggered mPFC stimulation prevents pathological cortical response. **a**, Schematic representation of CL intervention demonstrating IED_HC_ detection (scale bar, 1 s), responsively delivered waveform and refractory period between subsequent stimulations. Sample raw mPFC LFP trace after CL stimulation triggered on detected hippocampal IED (stimulation artifact occurs in region of orange dashed box; scale bar = 200 μV, 500 ms). **b**, Averaged spectrogram at time of IED_HC_ for a kindled-only (top) and a CL-stimulated (bottom) rat. Dotted box shows the cropped stimulation artifact (*n* = 1,000 IEDs from sample rat for each condition). **c**, Sample power spectrum of mPFC LFP following IED_HC_ (500 ms interval) in kindled-only and CL-stimulated rat (*n* = 1,000 IEDs from sample rat for each condition). Inset shows change in SPI_mPFC_ band power for kindled-only (*n* = 6 rats) and CL stimulation (from *n* = 11 rats; unpaired, two-tailed Mann–Whitney, *U* = 9,580, *P* = 1.15 × 10^−40^). *P < 0.05. **d**, Sample CCG of IED_HC_ with SPI_mPFC_ in a kindled-only rat (left; 8,223 IEDs and 9,974 spindles) and CL-stimulated rat (right; 6,687 IEDs and 5,475 spindles). The 95% confidence intervals with midpoints represented as black dashed and red lines. **e**, Histogram of all mPFC neuron firing after IED_HC_ for CL and kindled-only rats (left; CL = 714 neurons, seven rats; kindled-only = 2,733 neurons, six rats), putative pyramidal cells (middle; CL = 587 neurons, seven rats; kindled-only = 2,159 neurons, six rats) and putative interneurons (right; CL = 127 neurons, seven rats; kindled-only = 574 neurons, six rats). **f**, mPFC neural firing modulation after IED_HC_ in kindled-only and CL-stimulated rats (quantified during the 500 ms after cortical ‘DOWN’ state for kindled-only rats, and after the end of stimulation for CL-stimulated rats). Kruskal–Wallis with Dunn’s test, *χ*^2^ = 36.92—all neurons (*P* = 2.17 × 10^−4^, *n* = 72 and 30 sessions), pyramidal cells (*P* = 9.71 × 10^−4^, *n* = 72 and 30 sessions) and interneurons (*P* = 1, *n* = 48 and 27 sessions). *P < 0.05. Data are presented as mean ± s.e.m. in all panels. CL, closed loop; ALL, all neurons; PYR, pyramidal cells; INT, interneurons; NS, not significant. Source data

We designed a closed-loop electrical stimulation protocol aimed at preventing the hypersynchronous mPFC network response to hippocampal IEDs. We implanted rats with detection electrodes spanning the layers of hippocampal CA1 and stimulating electrodes capable of delivering bipolar electrical stimulation across layers of mPFC. Additional recording electrodes were present in mPFC to monitor the response to stimulation. These electrodes were integrated into an embedded system-based responsive device that was set for detection of hippocampal IEDs and delivery of responsive mPFC stimulation (Fig. 4a). Hippocampal IEDs were identified with sensitivity and specificity similar to conventional offline detection protocols^35,36^ (Supplementary Fig. 11). mPFC stimulation waveform was guided by evidence that spindles can be blocked by applying Gaussian waves across cortical layers, suggesting desynchronization of the mPFC network^35^. Closed-loop hippocampal IED-triggered mPFC stimulation resulted in a brief epoch (<1 s) characterized by a paucity of oscillatory waveforms in the physiological frequency band, followed by a recovery of prestimulation activity patterns (Fig. 4b,c). This closed-loop stimulation strongly inhibited spindles, as quantified by a significant decrease in spindle band power and IED–spindle cross-correlation compared to unstimulated rats (Fig. 4b–d).

We examined how our closed-loop stimulation protocol affected mPFC neural spiking patterns. Gaussian stimulation resulted in a significant decrease in subsequent population neural spiking compared to the ‘UP’ state following an unstimulated hippocampal IED. This change was mediated predominantly by pyramidal cells (Fig. 4e,f). Together, these results indicate that hippocampal IED-triggered closed-loop Gaussian stimulation can eliminate IED–spindle coupling by decreasing population neural firing.

### Closed-loop mPFC stimulation prevents memory deficits

Given the effectiveness of the closed-loop stimulation protocol in decoupling the mPFC from pathological hippocampal input, we investigated its network-level and behavioral outcomes. We studied the following three rat cohorts: (1) kindled-only, (2) sham stimulation and (3) closed-loop stimulation. All animals underwent the same kindling protocol (Methods), with equivalent efficacy in generation of IEDs across male and female rats (Supplementary Fig. 12). The closed-loop stimulation cohort received hippocampal IED-triggered mPFC Gaussian stimulation for ~7 h after each daily kindling session. The sham stimulation consisted of identical stimulation waveform properties and mean number of stimulations over the same daily duration delivered independently of hippocampal IED timing (Supplementary Fig. 13a). This stimulation did not induce characteristic mPFC activity patterns, but mPFC spindle activity was low in the epoch following stimulation (Supplementary Fig. 13b–d). Neither closed-loop nor sham stimulation caused behavioral state change or altered NREM sleep characteristics, but small changes in REM sleep activity were observed, consistent with involvement of mPFC in REM regulation^37^; Supplementary Fig. 14).

**Fig. 5:**
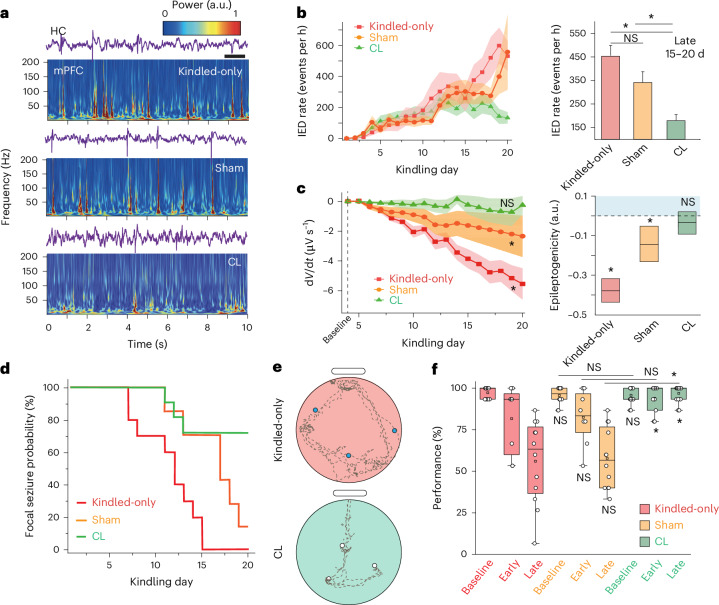
CL stimulation prevents epilepsy progression and memory deterioration. **a**, LFP traces from hippocampus and corresponding mPFC spectrograms for sample kindled-only, sham and CL rat. Scale bar, 1 s. **b**, IED_mPFC_ occurrence across kindling (left) and quantified at late kindling (right); ANOVA with Bonferroni–Holm correction, *P* = 2.93 × 10^−6^, *F* = 25.47; kindled-only/sham, *P* = 0.3287; CL/kindled-only; *P* = 2.64 × 10^−6^; CL/sham, *P* = 0.0049; kindled-only *n* = 39 sessions, 10 rats; sham *n* = 32 sessions, 7 rats; CL *n* = 48 sessions, 11 rats. *P < 0.05. **c**, mPFC epileptogenicity over kindling (left; Mann–Kendall tau; CL, *n* = 11 rats, *P* = 0.3; sham, *n* = 7 rats, *P* = 8.43 × 10^−8^; kindled-only, *n* = 10 rats, *P* = 8.42 × 10^−8^). Progression of epileptogenicity over kindling days for kindled-only and sham rats (right; linear mixed-effects model; CL, *P* = 0.188; sham, *P* = 0.001; kindled-only, *P* = 3.51 × 10^−26^). Box center = coefficient estimate, box boundaries = 5, 95% CI. *P < 0.05. **d**, Kaplan–Meier curve of progression from focal to bilateral convulsive seizures (kindled-only; *n* = 10 rats; sham, *n* = 7 rats; CL, *n* = 11 rats; log-rank test: kindled-only/sham, *P* = 1.25 × 10^−2^; kindled-only/CL, *P* = 2.42 × 10^−3^; sham/CL, *P* = 2.25 × 10^−2^). **e**, Examples of exploration path (dashed lines) with reward locations (blue circle, nonretrieved reward; open circle, retrieved reward) for the first three trials of the memory test in late kindling for sample kindled-only and CL rats. **f**, Memory performance over kindling for kindled-only (*n* = 4 rats), sham (*n* = 4 rats) and CL (*n* = 6 rats): ANOVA with Bonferroni–Holm correction: *F* = 19.76; kindled-only/sham: baseline (*P* = 0.77, *n* = 12/12 sessions), early (*P* = 0.90, *n* = 8/8 sessions) and late (*P* = 0.77, *n* = 12/12 sessions); sham/CL: baseline (*P* = 0.98, *n* = 12/17 sessions), early (*P* = 0.064, *n* = 8/11 sessions) and late (*P* = 4.15 × 10^−12^, *n* = 12/17 sessions); kindled-only/CL: baseline (*P* = 0.77, *n* = 12/17 sessions), early (*P* = 0.047, *n* = 8/11 sessions) and late (*P* = 7.85 × 10^−13^, *n* = 12/17 sessions). *P < 0.05. Data are presented as mean ± s.e.m. unless otherwise noted. Source data

Rats that underwent the closed-loop stimulation protocol exhibited significantly decreased development of mPFC IEDs compared to kindled-only and sham-stimulated animals (Fig. 5a,b and Supplementary Fig. 15a). This reduction occurred over the course of kindling, with the most profound differences observable during the late phase (Fig. 5a,b). A similar beneficial effect of the closed-loop stimulation on the occurrence of hippocampal IEDs was not observed (Supplementary Fig. 15c,d). Sham stimulation actually significantly increased hippocampal IED occurrence compared to kindled-only animals, although mild reductions in the rate of independent mPFC IEDs were observed with this protocol (Supplementary Fig. 15c,d). To further investigate interictal epileptogenicity of the mPFC, we designed an index that quantifies the sharpness and predictability of the LFP by examining its temporal first derivative (Supplementary Fig. 16). The closed-loop stimulation protocol was selectively effective in preventing an increase in interictal epileptogenicity (Fig. 5c). In parallel, we observed that rats undergoing the closed-loop stimulation protocol were significantly less likely to develop bilateral convulsive seizures during a duration of kindling that robustly generated this seizure semiology in control kindled rats. Sham stimulation delayed, but did not prevent, this progression (Fig. 5d and Supplementary Fig. 17a). These results suggest that closed-loop Gaussian mPFC stimulation triggered on hippocampal IEDs inhibits cortical recruitment into the mesial temporal epileptic network and preserves physiological properties of mPFC LFP.

We used a cheeseboard maze^38,39^ to assay the effect of our closed-loop stimulation protocol on memory. The behavioral protocol, consisting of a training session followed by a testing session the following day, was conducted before initiation of kindling, every 5 days during the kindling and after the kindling protocol was completed (Supplementary Fig. 17b). Rats were trained on the spatial location of three hidden water rewards, which were equivalently placed across cohorts (Supplementary Fig. 17c), and all animals were able to demonstrate effective learning by the end of the training session. On behavior training days, closed-loop or sham stimulation was initiated after the completion of training and continued for approximately 7 h post-training (Supplementary Fig. 10b). Kindled-only and sham animals displayed an early and progressive decrement of long-term memory performance as assayed by the ability to retrieve water rewards during the test trials. In contrast, rats who additionally underwent closed-loop stimulation maintained baseline high levels of memory performance (Fig. 5e,f and Supplementary Fig. 10d). Thus, this closed-loop network intervention can also preserve long-term memory capacity, indicating an overall protective effect on both mPFC network activity and function.

## Discussion

We demonstrate that temporally specific inhibition of pathological hippocampal–cortical oscillatory coupling prevents recruitment of synaptically connected cortex into the epileptic network and preserves long-term memory in a rodent focal epilepsy model. This closed-loop electrical stimulation intervention decreased hypersynchronous cortical neural spiking, blocking the prolonged and amplified response associated with uninterrupted IED–spindle coupling. IED–spindle coupling occurs in children and adults with focal epilepsy^24,25,35,40^, and we found a similar relationship between oscillatory coupling and independent IED foci in patients with epilepsy.

We found that interictal activity can contribute to a process that, over time, enlarges the brain territory capable of independently generating IEDs. When patients express IEDs outside of the seizure onset zone, the degree of IED independence varies, potentially indicating a similar longitudinal process. Epilepsy surgery has an increased likelihood of favorable outcome when the entire irritative zone is addressed, suggesting that impeding expansion of this zone could have therapeutic implications^41^. However, the inability to define the network organization of the irritative zone limits its utility in planning surgical resection and determining causal relationships to cognitive comorbidities^42–44^. Our results support that measures that inhibit the development of independent IED foci can prevent long-term memory deficits.

In our rodent model, the mPFC responded strongly and consistently to hippocampal IEDs by mounting an exaggerated version of the physiological reaction to hippocampal output^45^—a burst of cortical neural spiking followed by a ‘DOWN’ state and spindle oscillation (during the following ‘UP’ state). This responsiveness decreased as kindling progressed, paralleled by an increase in capacity for local hypersynchronous neural spiking and epileptic activity. In patients, a similar tradeoff revealed decreased IED–spindling coupling modulation associated with increased incidence of independent IEDs. Our observations of neural spiking activity suggest a pathological adaptation in epilepsy^46^ and could underpin the interictal network segregation identified by resting-state functional magnetic resonance imaging (MRI) in patients with progressive focal epilepsy^47^. The network mechanisms potentially responsible for this long-term restructuring of hippocampal–cortical interactions require additional exploration. Several mechanistic categories could be considered, including (1) synaptic plasticity and/or metaplasticity of the hippocampal–mPFC and intra-mPFC synapses, (2) changes in cell-type-specific microcircuit firing patterns that alter levels of cortical inhibition, (3) structural alterations in neuronal density or myelination and (4) modification of the neuromodulatory milieu (Supplementary Fig. 18). Hippocampus–mPFC synapses reliably express long-term potentiation, long-term depression (LTD) and depotentiation, with repetitive bursts of high-frequency stimulation, perhaps akin to ongoing IEDs, capable of inducing LTD^48–50^. Sustained LTD of hippocampal–mPFC synapses paired with a homeostatic strengthening of intracortical synapses could recapitulate the network patterns we observe. Similarly, cortical parvalbumin and somatostatin interneurons regulate feedforward inhibition and long-range synchrony between these regions, respectively, such that a cell-type-specific imbalance of firing could shift the effectiveness of inputs^51,52^. Although hippocampal kindling does not result in marked neuronal loss^53^, other microstructural alterations in the hippocampus and/or mPFC are likely. Myelin plasticity, which can be differentially activated in local and long-range white matter structures, could also have a role^54^. Finally, the mPFC is strongly affected by a variety of neuromodulators, changes in which could generate pathway-specific regulation^55–57^.

The closed-loop electrical stimulation protocol we used was designed and tested to eliminate pathological hippocampal–cortical coupling by preventing expression of a cortical ‘DOWN’ state and subsequent sleep spindle when delivered in response to a hippocampal IED. We affirmed the efficacy of this stimulation in eliminating IED–spindle coupling and further determined that it prevented the synchronized increase in population firing associated with the uninterrupted response of the mPFC network to hippocampal IEDs. When provided on an ongoing basis, such stimulation was capable of preventing the establishment of independent mPFC IEDs and normalizing mPFC network parameters, despite concurrent daily hippocampal seizures. These results suggest that hippocampal input in isolation is insufficient to drive long-lasting cortical network change; the subsequent cortical response is an integral contributor, potentially by instantiating local plasticity processes. Open-loop stimulation, which induced a similar transient decrease in population neural firing and was associated with low spindle band power, was capable of delaying seizure progression, but was ultimately less effective in modulating mPFC network activity than the closed-loop stimulation, and was unable to prevent memory deficits, in keeping with the notion that temporally targeted approaches can more sustainably modify networks^58^.

Because closed-loop electrical stimulation is clinically used in patients with epilepsy^59,60^, our approach has high translational potential—(1) it does not require viral vectors or genetic modifications, (2) the safety of electrical stimulation is established and (3) technological advancements can enhance computational capacity and decrease invasiveness of electronic devices^36,61^. Here we were limited in our ability to characterize the temporal determinants of the stimulation, including the duration of efficacy after stimulation was stopped, any state-dependent effects and whether a similar efficacy could be obtained if stimulation was initiated within an established epileptic network. Our findings could apply to clinical situations to prevent epileptogenesis after brain insult^62,63^ and support further investigation. In addition, we instituted our intervention at one key node of the memory network (mPFC), which was sufficient to rescue memory for the examined task. It is possible that it would be necessary to target different or multiple nodes to ensure intact memory across a range of hippocampus-dependent tasks.

Finely tuned and highly regulated hippocampal–cortical communication is required for multiple cognitive processes. Our results emphasize that hippocampal–cortical dynamics during the interictal state are modifiable targets to ameliorate epilepsy-associated memory dysfunction. We provide evidence for plasticity that is instantiated by chronic interictal epileptic activity patterns and eventually downregulates hippocampal input, at the expense of increased local mPFC hypersynchrony. Rebalancing the hippocampal–cortical interaction by inhibiting the pathological mPFC response may prevent this plasticity and preserve physiological mPFC activity patterns needed for memory consolidation. Thus, spatiotemporally targeted interventions that block the network effect of IEDs may modify disease course and ameliorate cognitive comorbidities in individuals with focal epilepsy.

## Methods

### Animal usage

Thirty-six male and female Long-Evans rats (200–350 g) underwent intracranial implantation and were distributed in the following cohorts: (1) 10 rats were kindled without additional electrical stimulation, (2) 11 rats had closed-loop stimulation during the kindling procedure, (3) 11 rats had sham stimulation during the kindling procedure, (4) 3 rats were used for induction of artificial IEDs and (5) 1 rat was used for bilateral hippocampus/mPFC implantation.

### Animal surgery procedure

All animal experiments were approved by the Institutional Animal Care and Use Committee at Columbia University Irving Medical Center. Rats were kept on a regular 12-h light/12-h dark cycle and housed in pairs before implantation but separated afterward. Prior experimentation was not performed on these animals, and experimentation was performed during light-on periods. The animals were initially anesthetized with 2% isoflurane and maintained under anesthesia with 0.75–1% isoflurane during surgery. Silicon probes (NeuroNexus) and/or 50 μm diameter tungsten wires mounted on custom-made micro-drives were implanted in the hippocampus (anterior–posterior (AP) = −3.5 and medial–lateral (ML) = 3.0) and ipsilateral mPFC (AP = 3.5, ML = 0.2 ML and dorsal–ventral (DV) = −2.5). Closed-loop and sham-stimulated rats were implanted with bipolar stimulation electrodes (50 μm diameter tungsten wires separated by 500 μm) spanning across mPFC cortical layers and in line with the recording electrodes. A pair of stimulating electrodes (two 50 μm diameter tungsten wires attached together with 500 μm dorsoventral tip separation) was implanted into the hippocampal commissure (AP = −0.5, ML = 0.8 ML and DV = −4.2) for electrical kindling stimulation or generation of a-IED_HC_. Screws in the skull, overlying the cerebellum, served as ground electrodes. The craniotomies were covered by Gelfoam and sealed using a 10:1 mixture of paraffin and mineral oil. Rats recovered for 4–5 days before initiation of further experimentation. Hippocampal electrodes were adjusted in the DV axis to span the layers of CA1 based on localizing neurophysiological signals.

### Kindling stimulation

Kindling stimulation consisted of 2 s duration bipolar current pulses (60 Hz, 1 ms pulse width) and was delivered twice per day (20 min separation interval between stimulations). The amount of current used was determined on the initial kindling days by titrating current in 5 μA increments starting at 25 μA (10 min separation interval between stimulations) until a hippocampal seizure greater than 20 s duration was generated. This current setting was used for the remainder of kindling. Five to seven hours of postkindling electrophysiological recordings were performed. No spontaneous seizures were detected in any rats using the following criteria to define electrographic seizures: high amplitude and rhythmic activity that evolves in amplitude and frequency with a duration of at least 5 s before offset.

### Closed-loop stimulation

Rats underwent the kindling procedure as previously described. Closed-loop stimulation consisted of 5–8 V Gaussian waves of 200 ms duration that were delivered across cortical layers. Stimulating electrode impedance was 20–50 kΩ, leading to an applied intracranial current of approximately 16–25 µA. Stimulation was adjusted to create Gaussian waves opposite in polarity to cortical ‘DOWN’ states, and voltage was titrated to the minimum required to suppress spindles. Stimulation was triggered by real-time detection of hippocampal IEDs using an individual rat-customized threshold on 50–85 Hz bandpass filtered data. Two rats had misplacement of mPFC stimulation electrodes and were removed from further experimentation. Starting 1 h after the second seizure induction, closed-loop stimulation commenced and was maintained for approximately 7 h per day.

### Sham stimulation

Stimulation was performed as described above, but cortical electrical stimulation was not coupled to the online detection of hippocampal IEDs. The frequency of stimulations corresponded to the average number of stimulations of closed-loop treated rats at the equivalent day of the kindling procedure.

### a-IED_HC_

Square pulses (200 μs) delivered to the hippocampal commissure were used for the induction of artificial IEDs. The stimulation voltage was adjusted to elicit hippocampal IEDs of amplitude comparable to that of spontaneous hippocampal IEDs in kindled rats (0.5–2.5 mV). Artificial IEDs were elicited every 3–5 s for 6–12 h per day. The protocol was administered daily with rare 1–2 day gaps. Spontaneous hippocampal IEDs were not detected. Independent IEDs were detected in the mPFC after 7–9 days of stimulation.

### Neurophysiological data acquisition and closed-loop system

Neurophysiological signals were amplified and digitized continuously at 20 kHz using a head-stage directly attached to the probe (Intan Technology, RHD2000) and stored for offline analysis with a 16-bit format (RHD USB Interface GUI version). For real-time IED detection and closed-loop stimulation, hippocampal data were routed via RHD2000 digital-to-analog converter into a custom 32-bit microcontroller (STM32)-based module. The data were filtered using an active bandpass filter (50–85 Hz). Subsequently, the filtered data were rectified and convolved with a moving average window to generate the instantaneous power of the signal at the frequency band of interest. The instantaneous power was then compared to the noise threshold, which was defined based on the s.d. of 10 s of baseline, filtered data. Surpassing the threshold triggered delivery of the preprogrammed cortical stimulation using a stimulus generator (Multichannel Systems, STG4002). Stimulation times were digitized and stored for offline analysis. To avoid subsequent delivery of stimulation during the period of network response, a refractory period for stimulation of 3 s was put in place. System parameters were visualized online via a graphical user interface, allowing for on-demand noise floor and stimulation threshold adjustment. Additionally, a three-axis accelerometer signal from the animal’s head-stage amplifier was continuously analyzed using a MATLAB custom algorithm to prevent stimulation triggered by any mechanical, movement-related artifacts.

### LFP preprocessing

Data were analyzed using MATLAB (2021b, MathWorks) and visualized using Neuroscope (http://sourceforge.net/projects/neuroscope). The electrophysiological data were resampled to 1,250 Hz to facilitate LFP analysis. Epochs of sleep were identified by immobility in the motion signal of the animal’s onboard accelerometer and absence of electromyogram artifacts. NREM and REM sleep epochs were classified using a validated automated sleep-scoring algorithm based primarily on ratios of cortical delta (0.5–4 Hz) and hippocampal theta (5–8 Hz)^64^. Sleep-scoring was visually inspected and manually adjusted if necessary using whitened spectrograms and raw traces. Activity patterns were detected using custom MATLAB code based on the Freely Moving Animal (http://fmatoolbox.sourceforge.net, v.20180316) toolbox during NREM sleep epochs. For data including stimulation epochs, stimulation artifacts were removed before analysis.

### Racine stages

Seizures induced by kindling were monitored by an overhead video camera. The severity of the seizures was scored according to Racine stages, which are as follows: stage 1, mouth and facial movements; stage 2, head nodding; stage 3, forelimb clonus; stage 4, rearing with forelimb clonus; and stage 5, rearing and falling with forelimb clonus. Stages 4 and 5 were considered as development of bilateral convulsive seizures.

### Human participants

We analyzed iEEG recordings from nine patients (male and female participants, aged 22–56 years) with focal epilepsy who underwent clinical electrode placement as part of the work-up for epilepsy surgery. The Institutional Review Board at New York University (NYU) Langone Medical Center approved the gathering and analysis of this data. Informed written consent was obtained from all patients. Patients were not compensated for participation. Patients were eligible if they were diagnosed with focal epilepsy, had continuous high-quality iEEG recordings, lacked major cortical lesions and had >1 clinically identified IED focus.

### Clinical reports

Clinical iEEG reports were obtained for each patient’s hospital admission, detailing localization of IEDs and the clinically identified seizure onset zone. Clinical interpretation was performed using a combination of referential montage (referenced to epidural electrodes) and bipolar montage (based on pairs of neighboring electrodes).

### iEEG data preprocessing and detections

Epochs of sleep were analyzed, and these were identified by immobility on synchronized video in concert with increased *δ*/*γ* frequency ratio in the iEEG spectrogram. Referential data was imported into MATLAB and resampled from 512 to 1,250 Hz for compatibility with previously validated analytical toolboxes. IED and spindle detection were performed on all electrodes as previously defined, in addition to IED–IED and IED–SPI coupling metrics^24^.

### iEEG electrode localization

Montreal Neurological Institute (MNI) coordinates of electrodes were determined by reconstruction of participant-specific pial surfaces, coregistration of preimplant and postimplant MRI images, a combination of manual and automatic localization of electrodes, and subsequent coregistration to a standard template brain^65^.

### IED detection

IEDs were detected by (1) bandpass filtering at 50–85 Hz and signal rectification, (2) detection of events for which the filtered envelope surpassed the median filtered signal by at least 5 s.d., (3) elimination of events for which the waveform amplitude (high-pass filtered above 15 Hz) did not surpass the mean baseline signal by at least 10 s.d. and (4) elimination of events for which the waveform amplitude surpassed the mean baseline by over 100 s.d. (consistent with artifact). Independence of IEDs was defined as the absence of a co-occurring IED in another brain region within 100 ms. Hippocampal IED detection was performed on the recording electrode with an average maximal IED amplitude deflection from baseline (generally located in CA1 stratum radiatum).

### Spindle detection

Cortical LFP was filtered between 10 and 20 Hz using a Butterworth filter. The filtered signal was then rectified, and instantaneous power was extracted using the Hilbert transform. Spindles were detected when the filtered envelope was at least 2 s.d. above the filtered baseline with an interposed peak at least 4 s.d., but not more than 14 s.d., above this baseline. The filtered baseline s.d. was calculated after epochs of cortical IEDs were removed. Additionally, spindle duration was defined as 350–3,000 ms, with detected events occurring within 250 ms merged into a single event.

### Cortical DOWN state detection

DOWN states were detected based on the identification of large positive deflections in the cortical LFP that were associated with decreases in the multiunit activity firing rate^66^. First, cortical LFP was filtered (0.5–6 Hz) and subsequently *z*-scored, which yielded *Z*(*t*). Next, the start (*t*_start_), peak (*t*_peak_) and end (*t*_end_) of putative DOWN states were defined as upward–downward–upward zero-crossings of the derivative of *Z*(t). Events with *Z*(*t*_peak_) > 1 and *Z*(*t*_end_) < −1.5 or *Z*(*t*_peak_) > 2 and *Z*(*t*_end_) < 0 were deemed candidate events. Finally, events with >500 ms or <150 ms durations were discarded. LFP-based DOWN state detection was validated by the instantaneous mPFC multiunit activity. Events where the multiunit activity decreased relative to *t*_peak_ were considered DOWN states. All detections were visually inspected for accuracy for each recording session. Pathological DOWN/UP transitions were classified as those that initiated within 200 ms of hippocampal IEDs. The remaining transitions were classified as physiological. The phase of the DOWN state in the delta band was derived using the Hilbert transformation of the filtered signal. For closed-loop and sham-stimulated rats, the stimulation artifacts (200 ms) were removed from the recordings before performing the corresponding event detections.

### Time domain cross-correlograms (CCGs) and coupling modulation

To determine coupling between detected oscillations, CCGs were calculated using a modified convolution method, as previously described^20,26,67^. The 95% confidence intervals were estimated from a Poisson distribution with the mean lambda value determined from the convolution. The peak of the CCG (*a*) above the 95% confidence interval and expected baseline value level (*b*) at time zero enabled calculation of the coupling modulation (*M*) as a normalized ratio—*M* = (*a* − *b*)/*b*. This approach takes into account the baseline occurrence rate of each LFP event to avoid spurious correlations.

### Frequency domain analysis

Spectrograms were generated using an analytical wavelet transformation (Gabor). Spindle band power was extracted from *z*-scored NREM power spectra. To compute the coherence between the hippocampus and mPFC, 10 s long LFP segments from baseline (that is, without kindling) and early and late kindling days across NREM, REM and wakefulness states were randomly selected. Coherence was calculated using the multitaper method in the Chronux toolbox (v.2.12 v03)^68^. The coherence was defined as follows:

$$C\left(\,f\,\right)=\frac{{S}_{12}(\,f\,)}{\sqrt{{S}_{11}(\,f\,){S}_{22}(\,f\,)}},$$

where *S*_12_(*f*) is the cross spectrum between the hippocampus and mPFC, *S*_11_ is the spectrum of the hippocampal LFP signal segment and *S*_22_ is the spectrum of the mPFC LFP signal segment. The absolute value of *C*(*f*) at various frequencies (1–100 Hz) generated the coherence spectrum, which was subsequently normalized.

### LFP epileptogenicity

To examine changes to NREM waveforms across kindling in a detection-free manner, segments of NREM sleep (duration = 5 s; *n* = 10 segments per session) were selected using a random number generator and with manual verification. Each segment was passed through a Savitzky–Golay filter to equalize the content of higher frequencies across segments. The gradient of each filtered sample was taken, and values greater than a noise level determined by a median-based threshold were removed. These threshold values were normalized by *z*-scoring relative to each rat’s baseline values across kindling days and were fit to a linear regression model to derive the slope of LFP changes and their statistical significance.

### Spiking-data processing

Noise-free epochs of data were used to first generate realistic neural spike templates and perform noise floor estimation. Multiunit activity was detected on the basis of spike amplitude using derivative-and-shift peak finding and median-based thresholding methods. Spike sorting was performed on bandpass filtered (250–2500 Hz) data using KiloSort (v.1.0)^69^. Manual cluster cutting and curation to segregate single neurons were performed using Phy (v.2.0). Single-units were further validated based on observation of mean waveform shape, auto-correlogram and consistency of the localization of the mean waveform with the probe geometry. Putative excitatory and inhibitory neurons were identified based on their auto-correlograms and waveform characteristics using CellExplorer (https://github.com/peterpetersen/CellExplorer)^34^.

### Neural spiking modulation measures

Zenith of event-based time-locked anomalies (ZETA; https://github.com/JorritMontijn/ZETA) was used to determine whether individual neurons showed a statistically significant time-dependent firing rate modulation relative to an event in a manner that avoids arbitrary parameter selection and binning^70^. ZETA identified neurons that showed significantly modulated spiking activity (*P* < 0.05) with respect to hippocampal and mPFC detected events and yielded instantaneous firing rate (IFR) amplitudes and latencies. These raw IFR peak values were divided by the baseline firing rate of each cell to quantify the normalized IFR modulation of each cell with respect to a reference event. IFR latency was computed by computing the temporal lag between the reference event and the IFR peak. Recording sessions containing less than 20 events were excluded from analysis. Peri-event time histograms of single neurons were normalized by the baseline firing activity before combining them across animals and kindling sessions. To ensure that observed single-unit activity measures were not driven by variability across animals, a generalized linear mixed-effects model was used with rat identity as a random effects term^71^. Only cells that were significantly modulated by the index event were included to generate the peri-event time histograms and comparison of neuronal modulation between kindling stages. Spike population rates were computed by summing all the detected single-unit activity with 1-ms resolution and smoothing the resulting population rate vector with a 50-ms Gaussian window. Normalization was performed by dividing the spiking population rate of each individual session by its respective baseline population firing rate values.

### Cheeseboard maze memory test

Rats were placed on a water deprivation schedule for 3–5 days before intracranial implantation to ensure they could receive water through a handheld syringe. Rats were weighed daily during water deprivation to ensure that body weight did not decrease to <85% of predeprivation measurements. Behavior for all tasks was tested on a cheeseboard maze as previously described^24^.

Before surgery, water-deprived rats were first familiarized with exploring the maze environment to obtain water. Initially, the rat was placed in the center of the maze and allowed to explore and retrieve multiple (~25) randomly placed hidden water rewards. Over the next 3 days, the number of available water rewards on the maze was gradually reduced, and a trial structure was introduced such that the rat received a food reward (0.5–1 Froot Loop) after successful retrieval of all water rewards. The rat was then trained to return to the starting box after retrieving water to obtain its food reward. After 2–4 days of this repeated procedure, the rat would consistently explore the maze to obtain three spatially distinct water rewards and then independently return to the starting box. To prevent the use of odor-mediated searching, the maze was wiped with a towel soaked in 70% ethanol and rotated by a random multiple of 90° relative to the starting box between all trials.

Each memory cycle on this task was completed over 2 days. On the first day, the rats learned the location of three hidden water rewards placed in a randomly selected set of three water wells over the course of ~40 trials (25 trial sessions, then ~3-h home cage rest and then 15 trial sessions). All rats obtained >90% performance averaged over five trials by the end of the training session. On the second day, the rat was given a three-trial test with water rewards located in the same location as the first day to assess memory for the spatial configuration of the reward locations.

Memory performance in the test session was scored by determining the percentage of rewards obtained per trial (performance percentage = number of retrieved rewards/total number of available rewards × 100). Behavior sessions were monitored by an overhead video camera and tracking of the rat’s location was facilitated by blue and red light-emitting diodes attached to its cap. Rats were trained to obtain >80% memory performance in the test session before intracranial electrode implantation. After the implantation and recovery, the rats were tested in three memory cycles before starting the kindling procedure to establish baseline memory performance. Subsequently, the rats were tested every 5 days during the kindling. Learning trials started at least 5 h after seizure induction, and test trials were performed before the subsequent day’s kindling. Closed-loop electrical stimulation was performed in the home cage after behavioral sessions. To ensure that observed memory performance was not driven by variability across animals, a generalized linear mixed-effects model was used with rat identity as a random effects term.

### Histology

After completion of experimentation, the placement of the implanted electrodes was verified for all the rats. Rats were killed with sodium pentobarbital and perfused via the heart with PBS followed by 4% paraformaldehyde (PFA). Whole brains were extracted and postfixated for 48 h in 4% PFA, embedded in 5% agar and sectioned using a VT-1000S vibratome (Leica) to obtain 70 μm coronal slices. Slices were permeabilized in PBS with Triton X-100 0.25%, stained with DAPI (1:10,000) for 20 min and mounted with Fluoromount-G (Invitrogen). A fluorescence Revolve microscope (ECHO) was used to image the slices. Tungsten wires or probe locations were reconstructed from adjacent slices.

### Statistics

Statistical analysis was performed using a combination of freely available, online MATLAB toolboxes (Freely Moving Animal Toolbox; http://fmatoolbox.sourceforge.net, custom MATLAB code and Origin Pro version 2021). Normality was examined using the Kolmogorov–Smirnov test. Differences between groups were calculated using *t*-test or analysis of variance (ANOVA; with Bonferroni–Holm correction for multiple comparisons) for normally distributed samples, and Mann–Whitney test or Kruskal–Wallis test (Dunn’s post hoc test) for samples derived from non-normal distributions, depending on the nature of the data analyzed. All tests were unpaired and two-tailed unless otherwise noted. Mann–Kendall tau was used to identify significant longitudinal trends. Error bars represent mean ± s.e.m. Significance level was *P* < 0.05. Box and whisker plots are defined as follows: centerline = median; box borders = lower and upper quartiles; whiskers = 5th and 95th percentiles.

### Reporting summary

Further information on research design is available in the Nature Portfolio Reporting Summary linked to this article.

## Online content

Any methods, additional references, Nature Portfolio reporting summaries, source data, extended data, supplementary information, acknowledgements, peer review information; details of author contributions and competing interests; and statements of data and code availability are available at 10.1038/s41593-025-01988-1.

## Supplementary information

Supplementary Information Supplementary Figs. 1–18.

Reporting Summary

## Source data

Source Data Fig. 1 Statistical source data.

Source Data Fig. 2 Statistical source data.

Source Data Fig. 3 Statistical source data.

Source Data Fig. 4 Statistical source data.

Source Data Fig. 5 Statistical source data.

## Data Availability

All data needed to evaluate the conclusions in the paper are present in the paper and/or the Supplementary Information. Relevant animal data are available through Mendeley Data (10.17632/2mpv3whxvs.1). Human participant data are subject to a data use agreement with NYU Langone Health. Requests pertaining to human participant data may be submitted to the corresponding authors with the expectation of a timely response. Source data are provided with this paper.

## Code availability

Relevant code is available through Mendeley Data (10.17632/2mpv3whxvs.1).

## References

[1] Lagarde, S. et al. Interictal stereotactic-EEG functional connectivity in refractory focal epilepsies. *Brain* **141**, 2966–2980 (2018). 30107499 10.1093/brain/awy214

[2] Hermann, B. P. et al. Cognitive prognosis in chronic temporal lobe epilepsy. *Ann. Neurol.* **60**, 80–87 (2006). 16802302 10.1002/ana.20872

[3] Kwan, P. & Brodie, M. J. Early identification of refractory epilepsy. *N. Engl. J. Med.* **342**, 314–319 (2000). 10660394 10.1056/NEJM200002033420503

[4] Perrine, K. et al. The relationship of neuropsychological functioning to quality of life in epilepsy. *Arch. Neurol.* **52**, 997–1003 (1995). 7575228 10.1001/archneur.1995.00540340089017

[5] Boylan, L. S. et al. Depression but not seizure frequency predicts quality of life in treatment-resistant epilepsy. *Neurology* **62**, 258–261 (2004). 14745064 10.1212/01.wnl.0000103282.62353.85

[6] Reed, C. M. et al. Extent of single-neuron activity modulation by hippocampal interictal discharges predicts declarative memory disruption in humans. *J. Neurosci.* **40**, 682–693 (2020). 31754015 10.1523/JNEUROSCI.1380-19.2019 PMC6961998

[7] Kleen, J. K. et al. Hippocampal interictal epileptiform activity disrupts cognition in humans. *Neurology* **81**, 18–24 (2013). 23685931 10.1212/WNL.0b013e318297ee50 PMC3770206

[8] Ortinski, P. & Meador, K. J. Cognitive side effects of antiepileptic drugs. *Epilepsy Behav.* **5**, 60–65 (2004). 10.1016/j.yebeh.2003.11.008 14725848

[9] Chvojka, J. et al. The role of interictal discharges in ictogenesis—a dynamical perspective. *Epilepsy Behav.* **121**, 106591 (2021). 31806490 10.1016/j.yebeh.2019.106591

[10] Dempsey, E. W. & Morison, R. S. The production of rhythmically recurrent cortical potentials after localized thalamic stimulation. *Am. J. Physiol.* **135**, 293–300 (1941).

[11] Chase, M. H., Nakamura, Y., Clemente, C. D. & Sterman, M. B. Afferent vagal stimulation: neurographic correlates of induced EEG synchronization and desynchronization. *Brain Res.* **5**, 236–249 (1967). 6033149 10.1016/0006-8993(67)90089-3

[12] Khodagholy, D., Ferrero, J. J., Park, J., Zhao, Z. & Gelinas, J. N. Large-scale, closed-loop interrogation of neural circuits underlying cognition. *Trends Neurosci.* **45**, 968–983 (2022). 36404457 10.1016/j.tins.2022.10.003 PMC10437206

[13] Jastrzebska‐Perfect, P. et al. Translational neuroelectronics. *Adv. Funct. Mater.* **30**, 1909165 (2020).

[14] Krook-Magnuson, E., Gelinas, J. N., Soltesz, I. & Buzsáki, G. Neuroelectronics and biooptics: closed-loop technologies in neurological disorders. *JAMA Neurol.* **72**, 823–829 (2015). 25961887 10.1001/jamaneurol.2015.0608 PMC4501886

[15] Morrell, M. J. Responsive cortical stimulation for the treatment of medically intractable partial epilepsy. *Neurology* **77**, 1295–1304 (2011). 21917777 10.1212/WNL.0b013e3182302056

[16] Bergey, G. K. et al. Long-term treatment with responsive brain stimulation in adults with refractory partial seizures. *Neurology* **84**, 810–817 (2015). 25616485 10.1212/WNL.0000000000001280 PMC4339127

[17] Toffa, D. H., Touma, L., El Meskine, T., Bouthillier, A. & Nguyen, D. K. Learnings from 30 years of reported efficacy and safety of vagus nerve stimulation (VNS) for epilepsy treatment: a critical review. *Seizure* **83**, 104–123 (2020). 33120323 10.1016/j.seizure.2020.09.027

[18] Chan, A. Y., Rolston, J. D., Rao, V. R. & Chang, E. F. Effect of neurostimulation on cognition and mood in refractory epilepsy. *Epilepsia Open* **3**, 18–29 (2018). 29588984 10.1002/epi4.12100 PMC5839311

[19] Bui, A. D. et al. Dentate gyrus mossy cells control spontaneous convulsive seizures and spatial memory. *Science* **359**, 787–790 (2018). 29449490 10.1126/science.aan4074 PMC6040648

[20] Khodagholy, D., Gelinas, J. N. & Buzsáki, G. Learning-enhanced coupling between ripple oscillations in association cortices and hippocampus. *Science* **358**, 369–372 (2017). 29051381 10.1126/science.aan6203 PMC5872145

[21] Siapas, A. G. & Wilson, M. A. Coordinated Interactions between hippocampal ripples and cortical spindles during slow-wave sleep. *Neuron* **21**, 1123–1128 (1998). 9856467 10.1016/s0896-6273(00)80629-7

[22] Girardeau, G. et al. Selective suppression of hippocampal ripples impairs spatial memory. *Nat. Neurosci.* **12**, 1222–1223 (2009). 19749750 10.1038/nn.2384

[23] Maingret, N., Girardeau, G., Todorova, R., Goutierre, M. & Zugaro, M. Hippocampo-cortical coupling mediates memory consolidation during sleep. *Nat. Neurosci.* **19**, 959–964 (2016). 27182818 10.1038/nn.4304

[24] Gelinas, J. N., Khodagholy, D., Thesen, T., Devinsky, O. & Buzsáki, G. Interictal epileptiform discharges induce hippocampal–cortical coupling in temporal lobe epilepsy. *Nat. Med.* **22**, 641–648 (2016). 27111281 10.1038/nm.4084 PMC4899094

[25] Yu, H. et al. Interaction of interictal epileptiform activity with sleep spindles is associated with cognitive deficits and adverse surgical outcome in pediatric focal epilepsy. *Epilepsia* **65**, 190–203 (2024). 37983643 10.1111/epi.17810 PMC10873110

[26] Dahal, P. et al. Interictal epileptiform discharges shape large-scale intercortical communication. *Brain* **142**, 3502–3513 (2019). 31501850 10.1093/brain/awz269 PMC6821283

[27] Sákovics, A. et al. Prolongation of cortical sleep spindles during hippocampal interictal epileptiform discharges in epilepsy patients. *Epilepsia* **63**, 2256 (2022). 35723195 10.1111/epi.17337 PMC9796153

[28] Buzsáki, G. Hippocampal sharp wave-ripple: a cognitive biomarker for episodic memory and planning. *Hippocampus* **25**, 1073–1188 (2015). 26135716 10.1002/hipo.22488 PMC4648295

[29] Shatskikh, T. N., Raghavendra, M., Zhao, Q., Cui, Z. & Holmes, G. L. Electrical induction of spikes in the hippocampus impairs recognition capacity and spatial memory in rats. *Epilepsy Behav.* **9**, 549–556 (2006). 17027341 10.1016/j.yebeh.2006.08.014

[30] Bartolomei, F. et al. Defining epileptogenic networks: contribution of SEEG and signal analysis. *Epilepsia* **58**, 1131–1147 (2017). 28543030 10.1111/epi.13791

[31] Sheybani, L. et al. Wake slow waves in focal human epilepsy impact network activity and cognition. *Nat. Commun.* **14**, 7397 (2023). 38036557 10.1038/s41467-023-42971-3 PMC10689494

[32] Sheybani, L. et al. Slow oscillations open susceptible time windows for epileptic discharges. *Epilepsia* **62**, 2357 (2021). 34338315 10.1111/epi.17020 PMC9290693

[33] Okadome, T. et al. The effect of interictal epileptic discharges and following spindles on motor sequence learning in epilepsy patients. *Front. Neurol.* **13**, 979333 (2022). 36438951 10.3389/fneur.2022.979333 PMC9686303

[34] Petersen, P. C., Siegle, J. H., Steinmetz, N. A., Mahallati, S. & Buzsáki, G. CellExplorer: a framework for visualizing and characterizing single neurons. *Neuron* **109**, 3594–3608 (2021). 34592168 10.1016/j.neuron.2021.09.002 PMC8602784

[35] Zhao, Z., Cea, C., Gelinas, J. N. & Khodagholy, D. Responsive manipulation of neural circuit pathology by fully implantable, front-end multiplexed embedded neuroelectronics. *Proc. Natl Acad. Sci. USA* **118**, e2022659118 (2021). 33972429 10.1073/pnas.2022659118 PMC8157942

[36] Cea, C. et al. Enhancement-mode ion-based transistor as a comprehensive interface and real-time processing unit for in vivo electrophysiology. *Nat. Mater.* **19**, 679–686 (2020). 32203456 10.1038/s41563-020-0638-3

[37] Hong, J., Lozano, D. E., Beier, K. T., Chung, S. & Weber, F. Prefrontal cortical regulation of REM sleep. *Nat. Neurosci.* **26**, 1820–1832 (2023). 37735498 10.1038/s41593-023-01398-1

[38] Roux, L., Hu, B., Eichler, R., Stark, E. & Buzsáki, G. Sharp wave ripples during learning stabilize the hippocampal spatial map. *Nat. Neurosci.* **20**, 845–853 (2017). 28394323 10.1038/nn.4543 PMC5446786

[39] Dupret, D., O’Neill, J., Pleydell-Bouverie, B. & Csicsvari, J. The reorganization and reactivation of hippocampal maps predict spatial memory performance. *Nat. Neurosci.* **13**, 995 (2010). 20639874 10.1038/nn.2599 PMC2923061

[40] Dahal, P., Rauhala, O. J., Khodagholy, D. & Gelinas, J. N. Hippocampal–cortical coupling differentiates long-term memory processes. *Proc. Natl Acad. Sci. USA* **120**, e2207909120 (2023). 36749719 10.1073/pnas.2207909120 PMC9963434

[41] Bautista, R. E. D., Cobbs, M. A., Spencer, D. D. & Spencer, S. S. Prediction of surgical outcome by interictal epileptiform abnormalities during intracranial EEG monitoring in patients with extrahippocampal seizures. *Epilepsia* **40**, 880–890 (1999). 10403211 10.1111/j.1528-1157.1999.tb00794.x

[42] Hufnagel, A., Diimpelmann, M., Zentner, T. J. & Elger, C. E. Clinical relevance of quantified intracranial interictal spike activity in presurgical evaluation of epilepsy. *Epilepsia* **41**, 467–478 (2000). 10756415 10.1111/j.1528-1157.2000.tb00191.x

[43] Janca, R. et al. The sub-regional functional organization of neocortical irritative epileptic networks in pediatric epilepsy. *Front. Neurol.* **9**, 184 (2018). 29628910 10.3389/fneur.2018.00184 PMC5876241

[44] Sabolek, H. R. et al. A candidate mechanism underlying the variance of interictal spike propagation. *J. Neurosci.* **32**, 3009–3021 (2012). 22378874 10.1523/JNEUROSCI.5853-11.2012 PMC3319688

[45] Wierzynski, C. M., Lubenov, E. V., Gu, M. & Siapas, A. G. State-dependent spike-timing relationships between hippocampal and prefrontal circuits during sleep. *Neuron* **61**, 587–596 (2009). 19249278 10.1016/j.neuron.2009.01.011 PMC2701743

[46] Issa, N. P., Nunn, K. C., Wu, S., Haider, H. A. & Tao, J. X. Putative roles for homeostatic plasticity in epileptogenesis. *Epilepsia* **64**, 539–552 (2023). 36617338 10.1111/epi.17500 PMC10015501

[47] Lucas, A. et al. Resting state functional connectivity demonstrates increased segregation in bilateral temporal lobe epilepsy. *Epilepsia* **64**, 1305–1317 (2023). 36855286 10.1111/epi.17565 PMC11934684

[48] Takita, M., Izaki, Y., Jay, T. M., Kaneko, H. & Suzuki, S. S. Induction of stable long-term depression in vivo in the hippocampal–prefrontal cortex pathway. *Eur. J. Neurosci.* **11**, 4145–4148 (1999). 10583503 10.1046/j.1460-9568.1999.00870.x

[49] Burette, F., Jay, T. M. & Laroche, S. Reversal of LTP in the hippocampal afferent fiber system to the prefrontal cortex in vivo with low-frequency patterns of stimulation that do not produce LTD. *J. Neurophysiol.* **78**, 1155–1160 (1997). 9307143 10.1152/jn.1997.78.2.1155

[50] Laroche, S., Jay, T. M. & Thierry, A. M. Long-term potentiation in the prefrontal cortex following stimulation of the hippocampal CA1/subicular region. *Neurosci. Lett.* **114**, 184–190 (1990). 2395531 10.1016/0304-3940(90)90069-l

[51] Abbas, A. I. et al. Somatostatin interneurons facilitate hippocampal-prefrontal synchrony and prefrontal spatial encoding. *Neuron* **100**, 926–939 (2018). 30318409 10.1016/j.neuron.2018.09.029 PMC6262834

[52] Marek, R. et al. Hippocampus-driven feed-forward inhibition of the prefrontal cortex mediates relapse of extinguished fear. *Nat. Neurosci.* **21**, 384–392 (2018). 29403033 10.1038/s41593-018-0073-9 PMC5957529

[53] Mathern, G. W. & Bertram, E. H. Recurrent limbic seizures do not cause hippocampal neuronal loss: a prolonged laboratory study. *Neurobiol. Dis.* **148**, 105183 (2021). 33207277 10.1016/j.nbd.2020.105183 PMC7855788

[54] Bonetto, G., Belin, D. & Káradóttir, R. T. Myelin: a gatekeeper of activity-dependent circuit plasticity? *Science* **374**, eaba6905 (2021). 34618550 10.1126/science.aba6905

[55] Ohashi, S., Matsumoto, M., Togashi, H., Ueno, K. I. & Yoshioka, M. The serotonergic modulation of synaptic plasticity in the rat hippocampo–medial prefrontal cortex pathway. *Neurosci. Lett.* **342**, 179–182 (2003). 12757894 10.1016/s0304-3940(03)00293-3

[56] Stoiljkovic, M., Kelley, C., Nagy, D., Hurst, R. & Hajós, M. Activation of α7 nicotinic acetylcholine receptors facilitates long-term potentiation at the hippocampal–prefrontal cortex synapses in vivo. *Eur. Neuropsychopharmacol.* **26**, 2018–2023 (2016). 27866776 10.1016/j.euroneuro.2016.11.003

[57] Park, A. J. et al. Reset of hippocampal–prefrontal circuitry facilitates learning. *Nature* **591**, 615–619 (2021). 33627872 10.1038/s41586-021-03272-1 PMC7990705

[58] Takeuchi, Y. & Berényi, A. Oscillotherapeutics—time-targeted interventions in epilepsy and beyond. *Neurosci. Res.* **152**, 87–107 (2020). 31954733 10.1016/j.neures.2020.01.002

[59] Lee, B. et al. A single-center experience with the NeuroPace RNS System: a review of techniques and potential problems. *World Neurosurg.* **84**, 719–726 (2015). 25940211 10.1016/j.wneu.2015.04.050

[60] Heck, C. N. et al. Two-year seizure reduction in adults with medically intractable partial onset epilepsy treated with responsive neurostimulation: final results of the RNS System Pivotal trial. *Epilepsia* **55**, 432–441 (2014). 24621228 10.1111/epi.12534 PMC4233950

[61] Hassan, A. R. et al. Translational organic neural interface devices at single neuron resolution. *Adv. Sci.* **9**, e2202306 (2022). 10.1002/advs.202202306 PMC9507374 35908811

[62] Schiff, N. D. et al. Thalamic deep brain stimulation in traumatic brain injury: a phase 1, randomized feasibility study. *Nat. Med.* **29**, 3162–3174 (2023). 38049620 10.1038/s41591-023-02638-4 PMC11087147

[63] Murphy, T. H. & Corbett, D. Plasticity during stroke recovery: from synapse to behaviour. *Nat. Rev. Neurosci.* **10**, 861–872 (2009). 19888284 10.1038/nrn2735

[64] Grosmark, A. D. et al. Reorganizes hippocampal excitability. *Neuron* **75**, 1001–1007 (2012). 22998869 10.1016/j.neuron.2012.08.015 PMC3608095

[65] Yang, A. I. et al. Localization of dense intracranial electrode arrays using magnetic resonance imaging. *Neuroimage* **63**, 157 (2012). 22759995 10.1016/j.neuroimage.2012.06.039 PMC4408869

[66] Todorova, R. & Zugaro, M. Isolated cortical computations during delta waves support memory consolidation. *Science* **366**, 377–381 (2019). 31624215 10.1126/science.aay0616

[67] Stark, E. & Abeles, M. Unbiased estimation of precise temporal correlations between spike trains. *J. Neurosci. Methods* **179**, 90–100 (2009). 19167428 10.1016/j.jneumeth.2008.12.029

[68] Bokil, H., Andrews, P., Kulkarni, J. E., Mehta, S. & Mitra, P. P. Chronux: a platform for analyzing neural signals. *J. Neurosci. Methods* **192**, 146–151 (2010). 20637804 10.1016/j.jneumeth.2010.06.020 PMC2934871

[69] Pachitariu, M. et al. Fast and accurate spike sorting of high-channel count probes with kilosort. In *Proc. Annual Conference on Neural Information Processing Systems (NeurIPS)* 4448–4456 (2016).

[70] Montijn, J. S. et al. A parameter-free statistical test for neuronal responsiveness. *eLife* **10**, e71969 (2021). 34570697 10.7554/eLife.71969 PMC8626082

[71] Yu, Z. et al. Beyond t test and ANOVA: applications of mixed-effects models for more rigorous statistical analysis in neuroscience research. *Neuron* **110**, 21–35 (2022). 34784504 10.1016/j.neuron.2021.10.030 PMC8763600

