# Supplementary material for: Closed-loop electrical stimulation prevents focal epilepsy progression and long-term memory impairment
Source: Nat Neurosci. 2025 Jun 23;28(8):1753–62. doi: 10.1038/s41593-025-01988-1 (PMC12321579; doi:10.1038/s41593-025-01988-1)
Supplement: Supplementary file 1 — Supplementary Figs. 1–18. [file 41593_2025_1988_MOESM1_ESM.pdf]

nature neuroscience

Supplementary information

https://doi.org/10.1038/s41593-025-01988-1

Closed-loop electrical stimulation prevents focal epilepsy progression and long-term memory impairment

In the format provided by the authors and unedited

Supplementary Materials for

Closed-loop electrical stimulation prevents focal epilepsy progression and long-term memory impairment

Jose J. Ferrero [†1, 2], Ahnaf R. Hassan [†1,2], Zelin Yu[3], Zifang Zhao[3], Liang Ma[1,2], Cynthia Wu[3], Shan Shao[1,2], Takeshi Kawano[4, 5], Judah Engel[3], Werner Doyle[6], Orrin Devinsky[6], Dion Khodagholy[3, 7,8]*, Jennifer N. Gelinas[1,2, 8, 9]*

1. Department of Neurology, Columbia University Irving Medical Center, New York, USA
2. Department of Pediatrics, University of California Irvine, California, USA
3. Department of Electrical Engineering, Columbia University, New York, USA
4. Department of Electrical and Electronic Information Engineering, Toyohashi University of Technology, Toyohashi, Japan
5. Institute for Research on Next-generation Semiconductor and Sensing Science (IRES2), Toyohashi University of Technology, Toyohashi, Japan
6. Comprehensive Epilepsy Center, New York University, New York, New York, USA.
7. Department of Electrical Engineering and Computer Science, University of California Irvine, California, USA
8. Department of Anatomy and Neurobiology, University of California Irvine, California, USA
9. Children's Hospital of Orange County, California, USA

†These authors contributed equally.

* Corresponding authors:
Dion Khodagholy: dion.kh@uci.edu
Jennifer Gelinas: gelinasj@hs.uci.edu

The PDF file includes:

Figs. S1 to S18

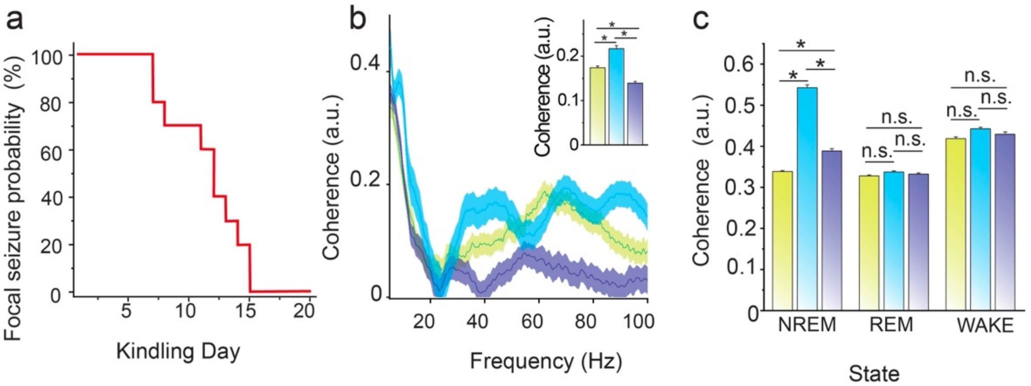

Supplementary Figure 1: Kindling protocol and state-dependent coherence.

(a) Progression from focal to bilateral convulsive seizures based on Racine stages, visualized using a survival curve (n = 10 rats).

(b) Coherence between hippocampus and mPFC during baseline (unkindled), early kindling, and late kindling with inset detailing differences in the 50-100 Hz frequency band (ANOVA with Bonferroni-Holm correction, F = 67.14: baseline vs early ($P = 7.44\times 10^{-13}$, n = 561 and 406 epochs); baseline vs late ($P = 7.36\times 10^{-6}$, n = 561 and 671 epochs); and early vs late ($P = 7.08 \times 10^{-30}$, n = 406 and 671 epochs); epochs sampled equivalently from 6 rats).

(c) Comparison of coherence at 50-100 Hz frequency band between hippocampus and mPFC during baseline (unkindled; green), early kindling (blue), and late kindling (purple) across behavior states. Kruskal-Wallis with Dunn's test, χ^2 =1485.71. NREM: baseline vs. early ($P = 1.33\times 10^{-4}$, n = 561 and 406 epochs); baseline vs. late ($P = 2.95\times 10^{-7}$, n = 561 and 671 epochs); and early vs. late ($P = 3.64 \times 10^{-22}$, n = 406 and 671 epochs). REM: baseline vs. early (P =1, n = 491 and 406 epoch); baseline vs. late (P = 1, n = 491 and 391 epochs); and early vs. late (P = 1, n = 438 and 391 epochs). WAKE: baseline vs. early (P = 0.16, n = 471 and 481 epochs); baseline vs. late (P = 1, n = 471 and 532 epochs); and early vs. late (P = 1, n = 481 and 532 epochs; all epochs sampled equivalently from n = 6 rats).

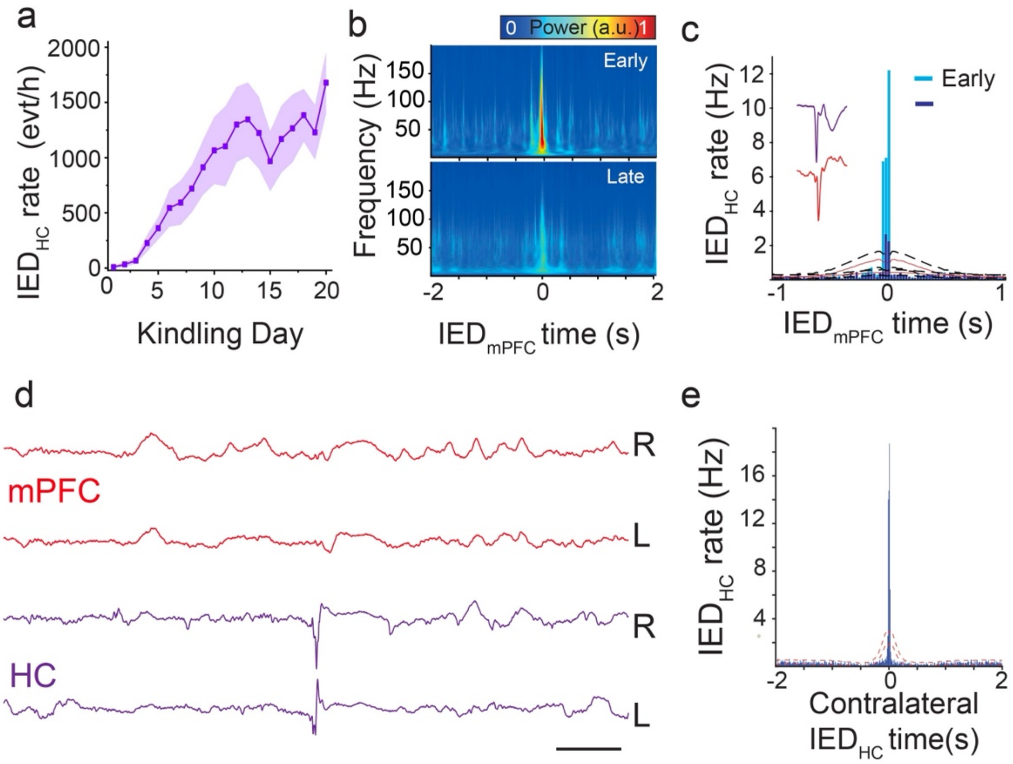

Supplementary Figure 2: **Emergence of independent mPFC IEDs.**

(a) Occurrence of hippocampal IEDs over kindling (n = 10 rats; shaded error bars represent SEM across rats).

(b) Averaged normalized spectrogram of hippocampal activity at the time of mPFC IEDs for early (upper) and late (lower) stages of kindling; 500 IEDs from 1 sample rat; IED power, early, 223 IEDs = 3.59 mV^2 and late, 552 IEDs = 0.97 mV^2 ($t = 16.6$, $P = 1.41 \times 10^{-41}$).

(c) Cross-correlograms of hippocampal and mPFC IEDs at early (blue; 557 IEDs and 11980 spindles) and late stages (purple; 1469 IEDs and 9026 spindles) of kindling from sample rat; 95% confidence intervals with midpoint represented as black dashed and red lines, respectively.

(d) Sample traces from bilateral mPFC (R = right; L = left) and bilateral hippocampi demonstrating synchronous occurrence of hippocampal IED; scale bar: 200 ms.

(e) Cross-correlogram of IEDs detected independently in right and left hippocampus during late kindling demonstrating bilateral synchrony (n = 2121 IEDs).

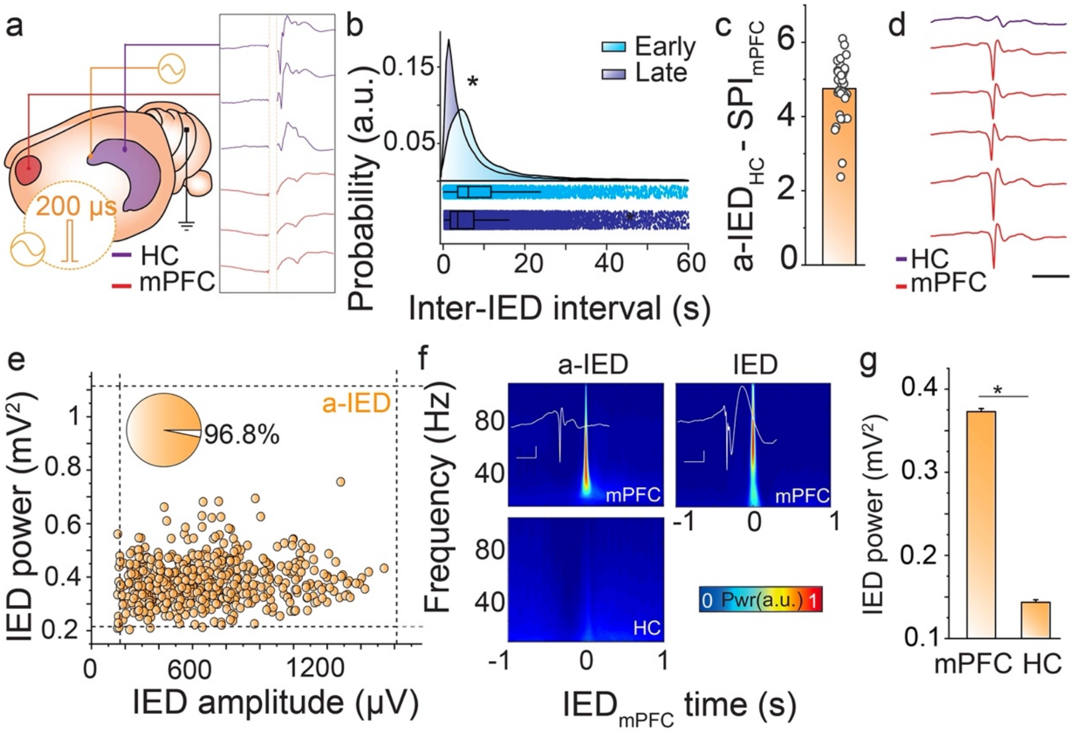

Supplementary Figure 3: Induction of artificial IEDs.

(a) Schematic of hippocampal commissure pulse stimulation (200 µs). Representative hippocampal LFP trace demonstrating artificial IED (a-IED$_{HC}$) and mPFC LFP trace showing evoked response.

(b) Inter-IED interval distributions for early (7956 IEDs) and late (41931 IEDs) kindling (two-sample Kolmogorov-Smirnov test, $P = 0$, test statistic = 0.265; n = 6 rats).

(c) Coupling modulation values ($M = (a-b)/b$) derived from cross-correlograms of a-IED$_{HC}$ and mPFC spindles (n = 35 sessions, from 3 rats).

(d) Induction of a-IED$_{HC}$ leads to development of independent mPFC IEDs in the absence of kindling. Averaged LFP traces (left) showing independent mPFC IED waveform across mPFC recording sites (red) in absence of hippocampal IED (purple; scale bar: 500 ms).

(e) Power and amplitude of independent mPFC IEDs generated by repetitive a-IED$_{HC}$ (orange circles, 595 IEDs from 3 rats). Dashed lines represent boundaries for mPFC IED detection in kindled rats (600 IEDs, 3 rats). Inset shows the percentage of independent mPFC IEDs (orange) with power and amplitude within range for detection if occurring in a kindled rat.

(f) Averaged spectrogram of mPFC (upper) and hippocampus (lower) at the time of independent mPFC IEDs generated by repetitive a-IED$_{HC}$ (left) or kindling-induced (right) mPFC IEDs. Superimposed white traces show trigger-averaged IED waveforms (n = 595 artificial IEDs from 3 rats, and 600 early IEDs from 3 kindled rats; scale bar: 200 ms, 200 µV).

(g) IED band power in mPFC and hippocampus at the time of independent mPFC IEDs generated by repetitive a-IED$_{HC}$ (595 artificial IEDs from 3 rats) revealing lack of co-occurring hippocampal IED (Mann-Whitney test, $U = 3.5 \times 10^{-5}$, $P = 0$).

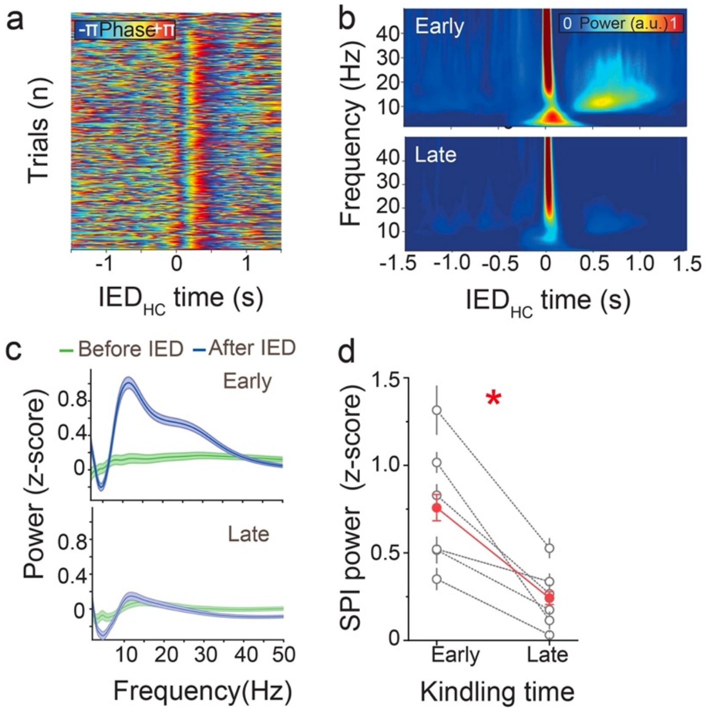

Supplementary Figure 4: Modulation of IED-spindle coupling across kindling.

(a) Stacked trials of mPFC delta phase (2-5 Hz) aligned to detection of hippocampal IED (blue = -pi, red = pi, n = 414 trials in one sample rat).

(b) Sample trigger-averaged spectrogram of mPFC power in early (upper) and late kindling (lower, n = 1000 IEDs).

(c) Sample power spectrum before (green; -1000 to -500 ms interval) and after (blue; 500 to 1000 ms interval) for early (upper) and late kindling (lower, n = 1000 randomly selected hippocampal IEDs in one rat; shaded bars are SEM).

(d) Decrease in spindle power from early to late stage of kindling across rats (n = 6 rats, comparing 24 sessions from early kindling to 24 sessions from late kindling, t = 6.17, P = 5.44 × 10^{-7}).

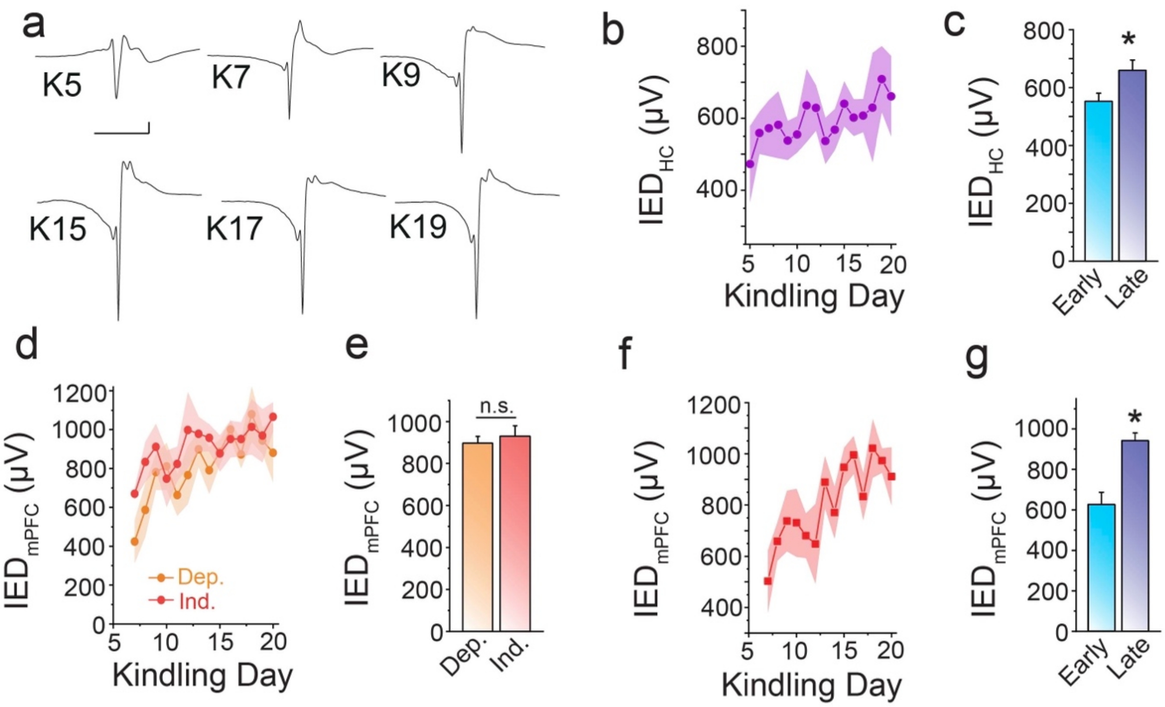

Supplementary Figure 5: Hippocampal and mPFC IED amplitudes across kindling.

(a) Average hippocampal IED waveforms over kindling days (K; n = 500 randomly selected IEDs for each session in one rat; scale bar: 200 µV, 100 µs).

(b) Amplitude of hippocampal IEDs across kindling (n = 6 rats; shaded error bars represent SEM across rats).

(c) Comparison of hippocampal IED amplitude during early and late kindling (t = -2.35, P = 0.024; n = 19 early sessions vs. 26 late sessions from 6 rats).

(d) Amplitude of hippocampal-dependent and independent mPFC IEDs across kindling (n = 6 rats, shaded error bars represent SEM across rats).

(e) Comparison of dependent and independent mPFC IED amplitude during late kindling (t = -0.55, P = 0.58; n = 21 early sessions vs. 26 late sessions from 6 rats).

(f) Amplitude of all mPFC IEDs across kindling (n = 6 rats, shaded error bars represent SEM across rats).

(g) Comparison of mPFC IED amplitude during early and late kindling ($t = -4.47$, $P = 8.65 \times 10^{-6}$; n = 20 early sessions vs. 25 late sessions from 6 rats).

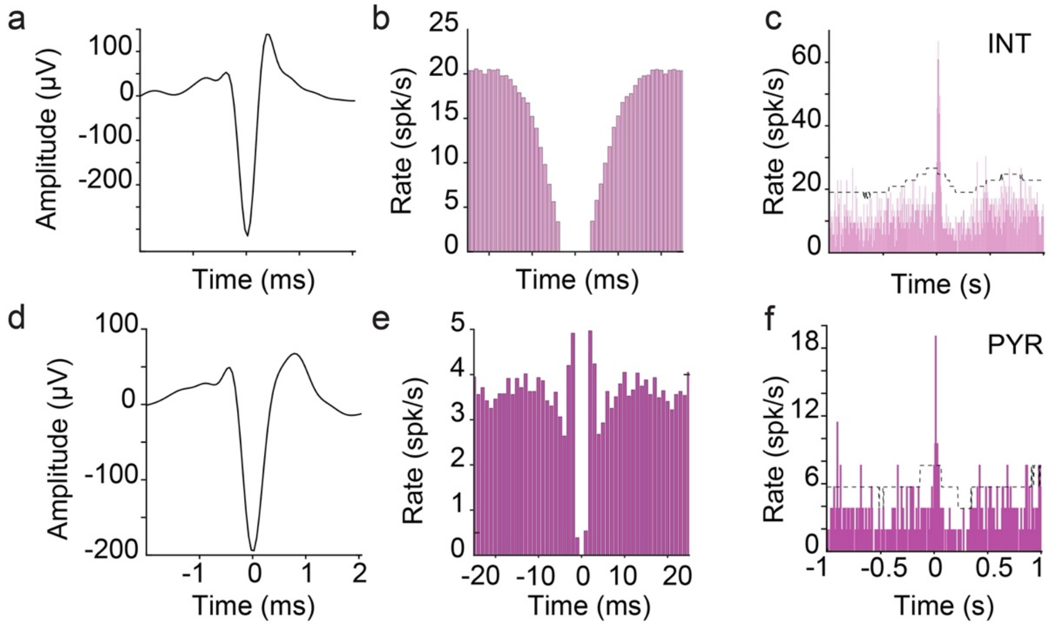

Supplementary Figure 6: Sample clustered units and response to hippocampal IEDs.

(a) Average clustered waveform of sample cortical interneuron.

(b) Autocorrelogram of sample cortical interneuron.

(c) Peri-event time histogram of neural spiking in response to hippocampal IED for sample cortical interneuron.

(d) Average clustered waveform of sample cortical pyramidal cell.

(e) Autocorrelogram of sample cortical pyramidal cell.

(f) Peri-event time histogram of neural spiking in response to hippocampal IED for sample cortical pyramidal cell.

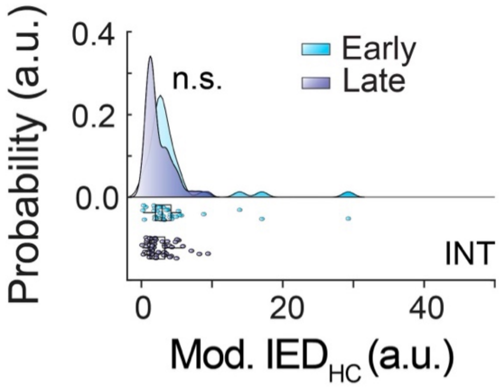

Supplementary Figure 7: mPFC interneuron spiking responses to hippocampal IEDs.

IFR peak probability distribution for clustered mPFC neurons at the time of hippocampal IEDs during early (blue) and late stages (purple) of the kindling for interneurons ($t = 1.93$, $P = 0.057$, early kindling $n = 41$; late kindling $n = 60$).

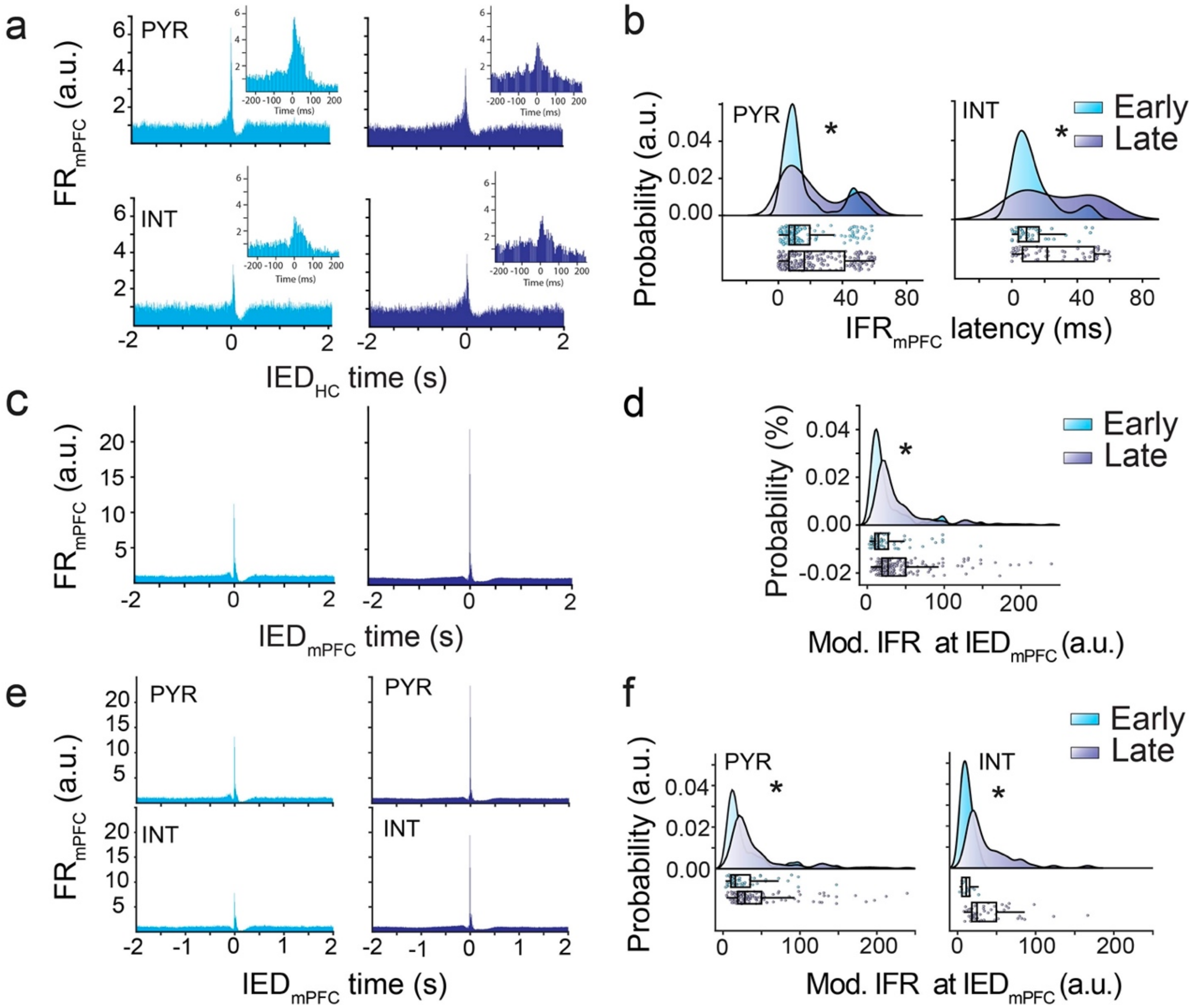

Supplementary Figure 8: mPFC neural spiking responses to hippocampal and mPFC IEDs.

(a) Averaged peri-event firing rate histogram of mPFC neurons at the time of hippocampal IEDs for early (left) and late (right) stage of kindling, for pyramidal cells (upper) and interneurons (lower). Insets show finer time-scale representations of each plot.

(b) mPFC neuron IFR latency probability distribution at the time of HC IEDs in early (blue) and late stages (purple) of the kindling, for pyramidal cells (left, $t = -2.91$, $P = 0.0039$, $n = 359$ neurons), and interneurons (right, $t = -3.68$, $P = 0.00037$, $n = 101$ neurons).

(c) Averaged peri-event firing rate histogram of mPFC neurons at the time of independent mPFC IEDs for early (left) and late stage of kindling (right).

(d) mPFC neural population IFR probability distribution at the time of independent mPFC IEDs in early (blue) and late stages (purple) of the kindling ($t = -2.11$, $P = 0.030$, $n = 268$ neurons).

(e) Averaged peri-event firing rate histogram of mPFC neurons at the time of independent mPFC IEDs in early (left) and late (right) stage of kindling, for pyramidal cells (upper) and interneurons (lower).

(f) mPFC neuron IFR probability distribution at the time of independent mPFC IEDs in early (blue) and late (purple) stages of kindling, for pyramidal cells (upper, $t = -2.30$, $P = 0.020$, $n = 195$ neurons) and interneurons (lower, $t = -3.21$, $P = 0.0020$, $n = 73$ neurons).

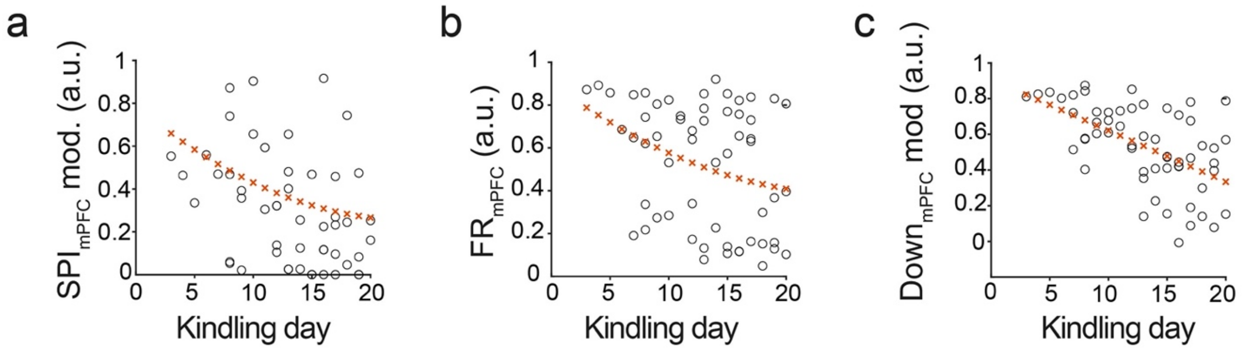

Supplementary Figure 9: mPFC oscillatory and neural spiking responsiveness decreases over kindling.

(a) Hippocampal IED-mPFC spindle coupling modulation decreases across kindling (n = 57 sessions from 6 rats).

(b) Hippocampal IED-evoked mPFC neural firing decreases across kindling (n = 57 sessions from 6 rats).

(c) Hippocampal IED-mPFC slow oscillation coupling modulation decreases across kindling (n = 57 sessions from 6 rats).

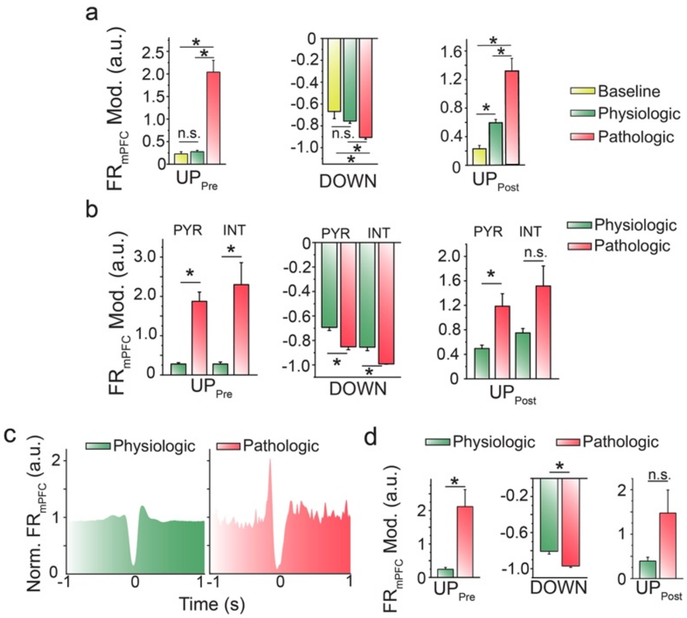

Supplementary Figure 10: mPFC pyramidal cell and interneuron firing during physiologic and pathologic cortical ‘UP’ and ‘DOWN’ states.

(a) Population firing rate modulation for transition through cortical ‘UP’ and ‘DOWN’ states for un-kindled rats (baseline, yellow) and kindled rats for physiological (green) and pathological (red) transitions: Kruskal-Wallis with Dunn’s test: UP_{pre} (left, averaged normalized values for 100 ms preceding cortical ‘DOWN’ state), $\chi^2 = 84.44$: baseline vs. physiologic, P = 1; baseline vs. pathologic, $P = 8.25 \times 10^{-5}$; physiologic vs. pathologic, $P = 1.60 \times 10^{-18}$. DOWN (middle, minimum normalized values for 200 ms following onset of cortical ‘DOWN’ state), $\chi^2 = 74.87$: baseline vs. physiologic, P = 0.52; baseline vs. pathologic, $P = 2.35 \times 10^{-6}$; physiologic vs. pathologic, $P = 3.03 \times 10^{-15}$. UP_{post} (right, averaged normalized values for 500 ms after peak of cortical ‘DOWN’ state), $\chi^2 = 28.14$: baseline vs. physiologic, P = 0.009; baseline vs. pathologic, $P = 1.44 \times 10^{-5}$; physiologic vs. pathologic, $P = 8.22 \times 10^{-4}$. n = 13 sessions for un-kindled rats and 118 sessions for kindled rats.

(b) Differences in mPFC pyramidal cell and interneuron firing rate modulation for physiological (green) and pathological (red) transition through cortical ‘UP’ and ‘DOWN’ states: UP_{pre} (left, averaged normalized values for 100 ms preceding cortical ‘DOWN’ state, pyramidal cells: Mann-Whitney test, U = 751, $P = 3.71 \times 10^{-40}$; interneurons: U = 409, $P = 4.10 \times 10^{-7}$), DOWN (middle, minimum normalized values for 200 ms following onset of cortical ‘DOWN’ state, pyramidal cells: U = 3775, $P = 1.33 \times 10^{-6}$; interneurons: U = 1779, $P = 1.90 \times 10^{-8}$), UP_{post} (right, averaged normalized values for 500 ms after peak of cortical ‘DOWN’ state, pyramidal cells: U = 1671, $P = 1.99 \times 10^{-4}$; interneurons: U = 918, P = 0.28; n = 118 sessions).

(c) Averaged peri-event firing rate histogram of mPFC neurons during physiological (green) and pathological (red) transition through cortical ‘UP’ and ‘DOWN’ states during kindling days prior to emergence of independent mPFC IEDs (n = 663 cells). Time 0 denotes maximal negativity of physiologic/pathologic cortical ‘DOWN’ state.

(d) Differences in population firing rate modulation for physiologic (green) and pathologic (red) transition through cortical ‘UP’ and ‘DOWN’ states during kindling days prior to emergence of independent mPFC IEDs: UP_{pre} (left, Mann-Whitney test, U = 4, $P = 1.03 \times 10^{-8}$), DOWN (middle, U = 268, $P = 2.77 \times 10^{-6}$), UP_{post} (right, U = 91, P = 0.067); n = 17 sessions.

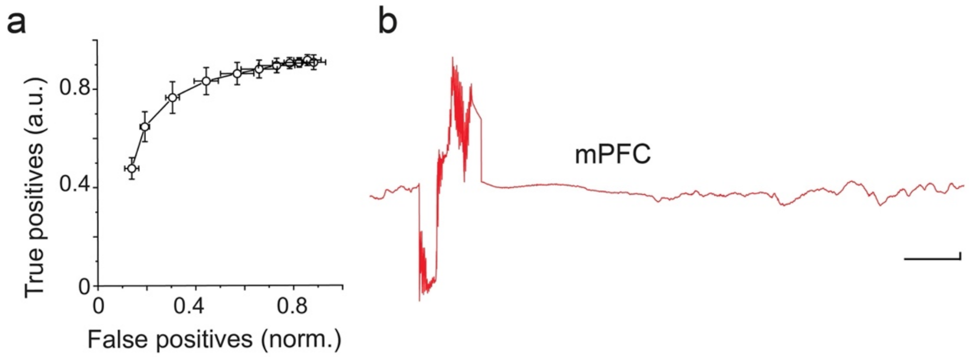

Supplementary Figure 11: Parameters of closed-loop stimulation protocol.

(a) Receiver operating characteristics curve for the online IED detection compared to the offline detection (n = 9946 IEDs, 5 sessions from 5 rats).

(b) Sample trace demonstrating stimulation artifact generated by electrical stimulation protocol (scale bar: 400 µA, 250 ms).

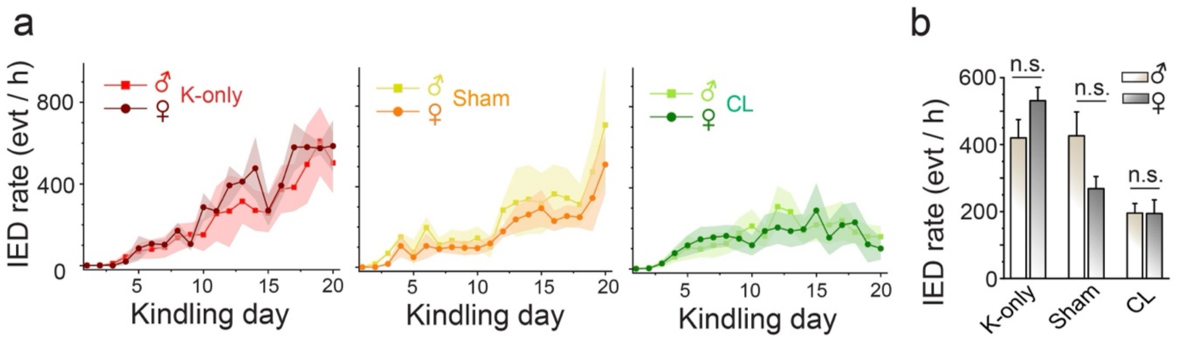

Supplementary Figure 12: Lack of sex-based differences in IED outcomes.

(a) Occurrence of total mPFC IEDs over kindling progression for female and male rats in kindled-only (left, n = 3 females and 7 males), sham (middle, n = 4 females and 3 males) and closed-loop stimulated (right, n = 6 females and 5 males) cohorts. Shaded error bars represent SEM across animals.

(b) Lack of difference in mPFC IED rate during late kindling between female and male rats ($t = -1.64$, $P = 0.11$; n = 13 and 34 sessions from 3 and 7 rats), sham ($t = 1.97$, $P = 0.06$; n = 22 and 17 sessions from 4 and 3 rats) and closed-loop ($t = 0.028$, $P = 0.97$; n = 29 and 30 sessions from 6 and 5 rats).

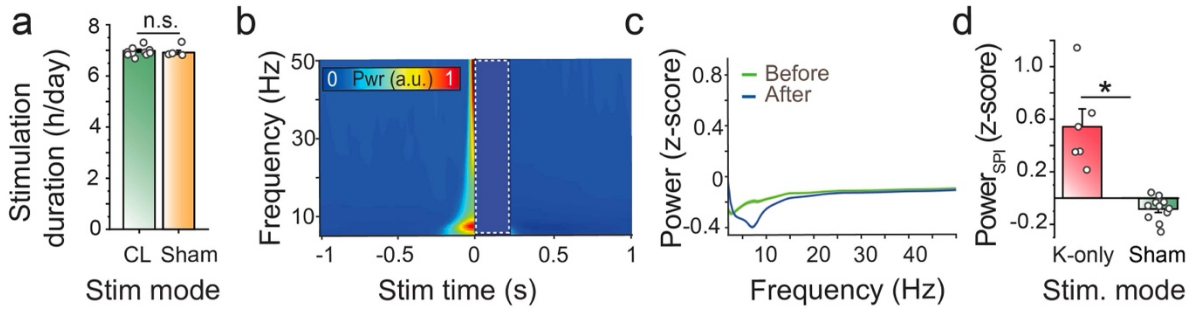

Supplementary Figure 13: Properties of closed-loop and sham stimulation protocols.

(a) Duration of stimulation treatment per day is equivalent between closed-loop (CL, n = 10 rats; green) and sham stimulated rats (n = 7 rats; blue); t = 0.69; P = 0.5017.

(b) Averaged mPFC spectrogram at the time of sham stimulation (n = 1000 randomly selected stimulations in one sample rat).

(c) Averaged mPFC power spectrum before (green, calculated from -1000 to -500 ms interval) and after (blue, 500 to 1000 ms interval) for sham stimulation (n = 1000 randomly selected stimulations in one sample rat).

(d) Change in mPFC spindle band power from before to after hippocampal IED in kindled-only rats (n = 6 rats) and sham stimulated rats (n = 7 rats, Mann-Whitney test, U = 9580, P = 1.15×10^{-40}).

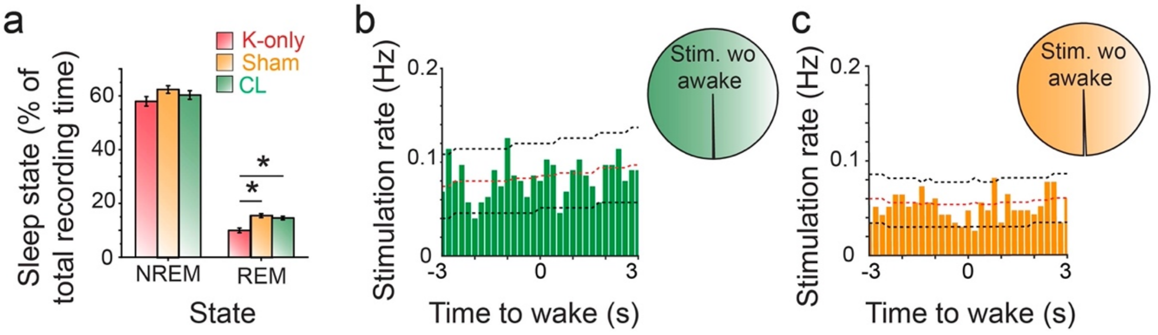

Supplementary Figure 14: mPFC electrical stimulation does not alter NREM sleep architecture or induce state change.

(a) NREM and REM sleep time as a percentage of the total duration of recording sessions. NREM: ANOVA with Bonferroni-Holm correction F = 2.54; kindling-only vs. sham, P = 0.2333; kindling-only vs. closed-loop, P = 0.2347; sham vs. closed-loop, P = 0.5562; REM: ANOVA with Bonferroni-Holm correction F = 11.11; kindling-only vs. sham, $P = 3.05 \times 10^{-4}$; kindling-only vs. closed-loop, P = 0.0038; sham vs. closed-loop, P = 0.5453; n = 10 kindled-only, 7 sham and 11 closed-loop stimulated rats.

(b) Cross-correlogram of mPFC electrical stimulations with transitions to wake state for closed-loop stimulation; 95% confidence intervals with midpoint represented as black dashed and red lines, respectively. Pie chart shows the percentage of stimulations not related to transitions to wake state (99.7% of 22280 stimulations, 876 wake transitions).

(c) Cross-correlogram of mPFC electrical stimulations with transitions to wake state for sham stimulation; 95% confidence intervals with midpoint represented as black dashed and red lines, respectively. Pie chart shows the percentage of stimulations not related to transitions to wake state (99.4% of 20397 stimulations, 1166 wake transitions).

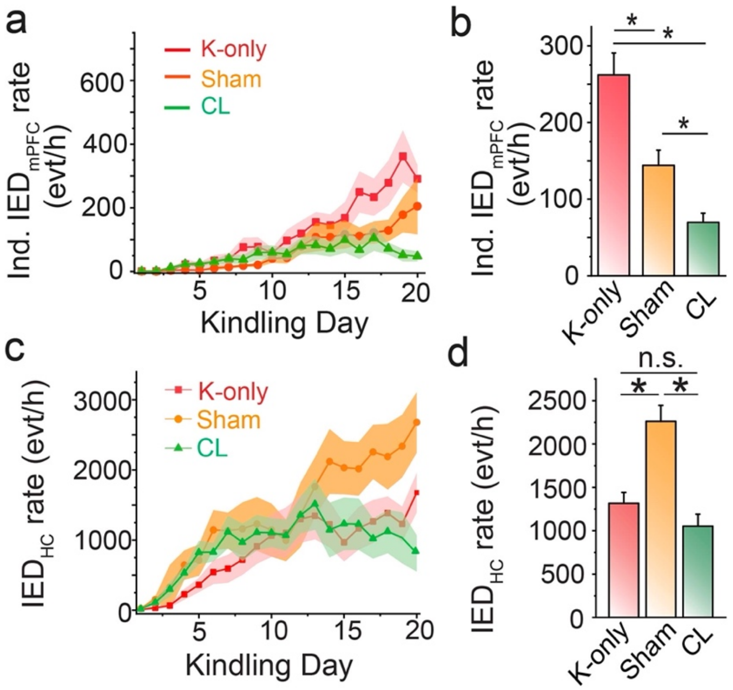

Supplementary Figure 15: Effects of stimulation on mPFC and hippocampal IED rates.

(a) Occurrence of independent IEDs in mPFC over kindling progression for kindled rats (left, n = 10 rats), sham (middle, n = 7 rats) or closed-loop stimulated (right, n = 11 rats, shaded error bars represent SEM).

(b) Quantification of independent mPFC IEDs at the late stages of kindling (days 15 – 20). ANOVA with Bonferroni-Holm correction, $F = 24.32$: kindled-only vs. sham ($P = 1.87 \times 10^{-4}$, n = 39 and 32 sessions from 10 and 7 rats); kindled-only vs. closed-loop ($P = 2.16 \times 10^{-10}$, n = 39 and 48 sessions from 10 and 11 rats); and sham vs. closed-loop ($P = 1.24 \times 10^{-2}$, n = 32 and 48 sessions from 7 and 11 rats).

(c) Occurrence of IEDs in hippocampus over kindling progression for kindled rats (left, n = 10 rats), sham (middle, n = 7 rats) or closed-loop stimulated rats (right, n = 11 rats, shaded error bars represent SEM).

(d) Quantification of hippocampal IEDs at the late stages of kindling (days 15 – 20). ANOVA with Bonferroni-Holm correction, $F = 16.87$: kindled-only vs. sham ($P = 4.22 \times 10^{-5}$, n = 40 and 32 sessions from 10 and 7 rats); kindled-only vs. closed-loop ($P = 0.19$, n = 40 and 49 sessions from 10 and 11 rats); and sham vs. closed-loop ($P = 9.43 \times 10^{-8}$, n = 32 and 49 sessions from 7 and 11 rats).

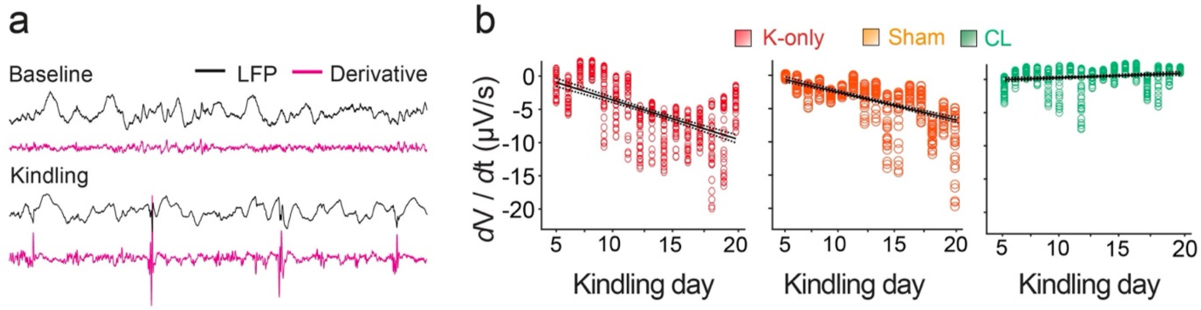

Supplementary Figure 16: Decrease in mPFC epileptogenicity with closed-loop stimulation.

(a) Sample first derivative trace (magenta) of wide-band mPFC LFP trace (black) prior to kindling (upper) and in kindled (lower) states.

(b) Variation in first derivative across kindling for sample kindled-only rat, sham stimulated rat, and closed-loop stimulated rat.

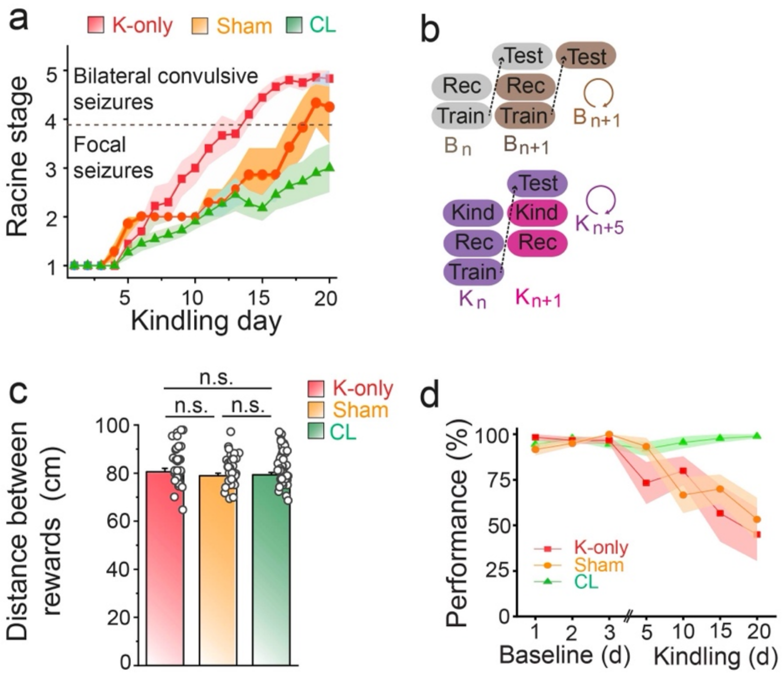

Supplementary Figure 17: Seizure progression and behavioral protocol.

(a) Racine stage progression over kindling for kindled-only rats (n = 10 rats), sham stimulated rats (n = 7 rats) and closed-loop stimulated rats (CL, n = 11 rats).

(b) Schematic of behavioral protocol during baseline (B, upper) and kindling (K, lower), showing the successive sessions during the memory training and testing: delivery of kindling stimulation (Kind), neurophysiological recording in absence or presence of closed-loop/sham stimulation (Rec), behavioral training (Train), and behavioral testing the subsequent day (Test).

(c) Averaged distance between rewards on the cheeseboard maze. ANOVA with Bonferroni-Holm correction, F = 0.52; kindled-only vs. sham (P = 0.34, n = 34 and 34 sessions from 4 and 4 rats); kindled-only vs. closed-loop (P = 0.42, n = 34 and 52 sessions from 4 and 6 rats); and sham vs. closed-loop (P = 0.81, n = 34 and 52 sessions from 4 and 6 rats).

(d) Memory test performance (percentage of rewards retrieved during five trials) before starting the kindling (baseline) and over kindling for kindled-only (n = 4 rats), sham (n = 4 rats), and closed-loop stimulated rats (n = 6 rats, shaded error bars represent SEM across rats; ANOVA with Bonferroni-Holm correction for kindling day 20, F = 11.15; kindled-only vs. sham (P = 0.56, n = 4 and 4 rats); kindled-only vs. closed-loop (P = 0.0014, n = 4 and 6 rats); and sham vs. closed-loop (P = 0.0042, n = 4 and 6 rats).

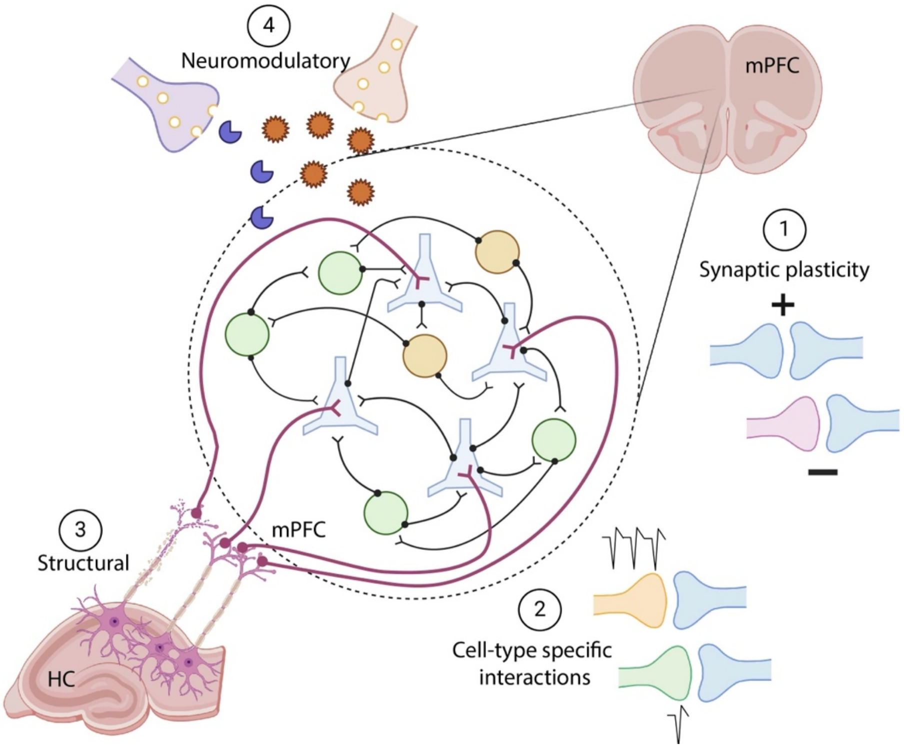

Supplementary Figure 18: Potential mechanisms underlying progressive restructuring of hippocampal-cortical interactions mediated by chronic epileptic activity.

(1) Synaptic plasticity and/or metaplasticity of the hippocampal-mPFC and intra-mPFC synapses.

(2) Changes in cell-type specific microcircuit firing patterns that alter levels of cortical inhibition.

(3) Structural alterations in neuronal density or myelination.

(4) Modification of the neuromodulatory milieu.
