# Supplementary material for: Closed-loop electrical stimulation prevents focal epilepsy progression and long-term memory impairment
Source: Nat Neurosci. 2025 Jun 23;28(8):1753–62. doi: 10.1038/s41593-025-01988-1 (PMC12321579; doi:10.1038/s41593-025-01988-1)
Supplement: Supplementary file 2 — Reporting Summary [file 41593_2025_1988_MOESM2_ESM.pdf]

nature portfolio

Corresponding author(s): Jennifer Gelinas, Dion Khodagholy

Last updated by author(s): 02/26/2025

Reporting Summary

Nature Portfolio wishes to improve the reproducibility of the work that we publish. This form provides structure for consistency and transparency in reporting. For further information on Nature Portfolio policies, see our Editorial Policies and the Editorial Policy Checklist.

Statistics

For all statistical analyses, confirm that the following items are present in the figure legend, table legend, main text, or Methods section.

n/a	Confirmed	
☐	☒	The exact sample size (*n*) for each experimental group/condition, given as a discrete number and unit of measurement
☐	☒	A statement on whether measurements were taken from distinct samples or whether the same sample was measured repeatedly
☐	☒	The statistical test(s) used AND whether they are one- or two-sided<br>*Only common tests should be described solely by name; describe more complex techniques in the Methods section.*
☐	☒	A description of all covariates tested
☐	☒	A description of any assumptions or corrections, such as tests of normality and adjustment for multiple comparisons
☐	☒	A full description of the statistical parameters including central tendency (e.g. means) or other basic estimates (e.g. regression coefficient) AND variation (e.g. standard deviation) or associated estimates of uncertainty (e.g. confidence intervals)
☐	☒	For null hypothesis testing, the test statistic (e.g. *F*, *t*, *r*) with confidence intervals, effect sizes, degrees of freedom and *P* value noted<br>*Give P values as exact values whenever suitable.*
☒	☐	For Bayesian analysis, information on the choice of priors and Markov chain Monte Carlo settings
☒	☐	For hierarchical and complex designs, identification of the appropriate level for tests and full reporting of outcomes
☒	☐	Estimates of effect sizes (e.g. Cohen's *d*, Pearson's *r*), indicating how they were calculated

Our web collection on statistics for biologists contains articles on many of the points above.

Software and code

Policy information about availability of computer code

Data collection	RHD 2000 Intan software (RHD USB Interface GUI version) was used for electrophysiological data acquisition.
Data analysis	MATLAB 2021b, Chronux toolbox (version 2.12 v03), FMA toolbox (version 20180316), Kilosort (version 1), Phy (version 2.0), CellExplorer (https://github.com/peterpetersen/CellExplorer), ZETA (https://github.com/JorritMontijn/ZETA), OriginPro (version 2021) were used for data analysis. Custom code utilized is available through Mendeley data repository.

For manuscripts utilizing custom algorithms or software that are central to the research but not yet described in published literature, software must be made available to editors and reviewers. We strongly encourage code deposition in a community repository (e.g. GitHub). See the Nature Portfolio guidelines for submitting code & software for further information.

Data

Policy information about availability of data

All manuscripts must include a data availability statement. This statement should provide the following information, where applicable:

- Accession codes, unique identifiers, or web links for publicly available datasets
- A description of any restrictions on data availability
- For clinical datasets or third party data, please ensure that the statement adheres to our policy

All data needed to evaluate the conclusions in the paper are present in the paper and/or the Supplementary Materials. Relevant animal data are available through

Mendeley Data (10.17632/2mpv3whxvs.1). Human subject data are subject to a data use agreement with NYU Langone Health. Requests pertaining to human subject data may be submitted to the corresponding authors with the expectation of timely response.

Research involving human participants, their data, or biological material

Policy information about studies with human participants or human data. See also policy information about sex, gender (identity/presentation), and sexual orientation and race, ethnicity and racism.

Reporting on sex and gender	Sex and gender of human participants was not relevant for this study and is not reported.
Reporting on race, ethnicity, or other socially relevant groupings	Race, ethnicity, and socially relevant groupings were not relevant for this study and are not reported.
Population characteristics	All human research participants underwent intracranial electroencephalographic monitoring for medically refractory epilepsy. Male and female subjects, aged 22-56 years, were included.
Recruitment	All patients undergoing intracranial electroencephalographic monitoring for medically refractory epilepsy are offered to participate in data collection. A chronologic subset of participants whose data met inclusion criteria were included. No systematic bias is expected due to these broad recruitment parameters and lack of need for direct subject participation in the research. Patients were not compensated for participation.
Ethics oversight	Institutional Review Board at NYU Langone Health

Note that full information on the approval of the study protocol must also be provided in the manuscript.

Field-specific reporting

Please select the one below that is the best fit for your research. If you are not sure, read the appropriate sections before making your selection.

☒ Life sciences ☐ Behavioural & social sciences ☐ Ecological, evolutionary & environmental sciences

For a reference copy of the document with all sections, see nature.com/documents/nr-reporting-summary-flat.pdf

Life sciences study design

All studies must disclose on these points even when the disclosure is negative.

Sample size	Resource equation method was used to estimate required sample size. This approach estimates required sample size when prior assumptions about effect size cannot be made, and multiple endpoints with independent statistical analysis are required for each animal's data.
Data exclusions	Data from rats was excluded if the intracranial implantation was not successful.
Replication	Reproducibility of the experimental findings was ensured by conducting experimentation with additional rats over the course of the study. Each animal experiment was performed independently and results were included in analyses except in the case of data exclusion as noted above.
Randomization	Rats were randomly assigned to control, sham, or closed-loop cohorts prior to experimentation. Human subject data was analyzed independently per subject, as these were observational analyses without experimentation performed.
Blinding	Blinding to group allocation was not possible because the intervention (electrical stimulation) caused identifiable artifacts in the electrophysiological data.

Reporting for specific materials, systems and methods

We require information from authors about some types of materials, experimental systems and methods used in many studies. Here, indicate whether each material, system or method listed is relevant to your study. If you are not sure if a list item applies to your research, read the appropriate section before selecting a response.

Materials & experimental systems

n/a	Involved in the study
☒	☐ Antibodies
☒	☐ Eukaryotic cell lines
☒	☐ Palaeontology and archaeology
☐	☒ Animals and other organisms
☒	☐ Clinical data
☒	☐ Dual use research of concern
☒	☐ Plants

Methods

n/a	Involved in the study
☒	☐ ChIP-seq
☒	☐ Flow cytometry
☒	☐ MRI-based neuroimaging

Animals and other research organisms

Policy information about studies involving animals; ARRIVE guidelines recommended for reporting animal research, and Sex and Gender in Research

Laboratory animals	Long Evans rats, 200-350g, 8-12 weeks of age
Wild animals	No wild animals were used
Reporting on sex	Rats of male and female sex were used. No sex-based differences in the electrophysiologic or behavioral parameters analyzed in this study were observed.
Field-collected samples	No field-collected samples.
Ethics oversight	Institutional Animal Care and Use Committee at Columbia University Irving Medical Center

Note that full information on the approval of the study protocol must also be provided in the manuscript.

Plants

Seed stocks	*Report on the source of all seed stocks or other plant material used. If applicable, state the seed stock centre and catalogue number. If plant specimens were collected from the field, describe the collection location, date and sampling procedures.*
Novel plant genotypes	*Describe the methods by which all novel plant genotypes were produced. This includes those generated by transgenic approaches, gene editing, chemical/radiation-based mutagenesis and hybridization. For transgenic lines, describe the transformation method, the number of independent lines analyzed and the generation upon which experiments were performed. For gene-edited lines, describe the editor used, the endogenous sequence targeted for editing, the targeting guide RNA sequence (if applicable) and how the editor was applied.*
Authentication	*Describe any authentication procedures for each seed stock used or novel genotype generated. Describe any experiments used to assess the effect of a mutation and, where applicable, how potential secondary effects (e.g. second site T-DNA insertions, mosiacism, off-target gene editing) were examined.*
